# Computational network analysis of host genetic risk variants of severe COVID-19

**DOI:** 10.1186/s40246-023-00454-y

**Published:** 2023-03-02

**Authors:** Sakhaa B. Alsaedi, Katsuhiko Mineta, Xin Gao, Takashi Gojobori

**Affiliations:** 1 Division of Computer, Electrical and Mathematical Sciences and Engineering, Computational Bioscience Research Center (CBRC), King Abdullah University of Science and Technology (KAUST), Thuwal, 23955-6900 Saudi Arabia; 2 College of Computer Science and Engineering (CCSE), Taibah University, Medina, Saudi Arabia; 3 AND Research Organization for Nano and Life Innovation, Waseda University, Tokyo, 162-0041 Japan

**Keywords:** Severe COVID-19, Host risk variants, GWAS, Genetic risk factor analysis, Molecular networks analysis, Disease mapping, Statistical analysis

## Abstract

**Background:**

Genome-wide association studies have identified numerous human host genetic risk variants that play a substantial role in the host immune response to SARS-CoV-2. Although these genetic risk variants significantly increase the severity of COVID-19, their influence on body systems is poorly understood. Therefore, we aim to interpret the biological mechanisms and pathways associated with the genetic risk factors and immune responses in severe COVID-19. We perform a deep analysis of previously identified risk variants and infer the hidden interactions between their molecular networks through disease mapping and the similarity of the molecular functions between constructed networks.

**Results:**

We designed a four-stage computational workflow for systematic genetic analysis of the risk variants. We integrated the molecular profiles of the risk factors with associated diseases, then constructed protein–protein interaction networks. We identified 24 protein–protein interaction networks with 939 interactions derived from 109 filtered risk variants in 60 risk genes and 56 proteins. The majority of molecular functions, interactions and pathways are involved in immune responses; several interactions and pathways are related to the metabolic and cardiovascular systems, which could lead to multi-organ complications and dysfunction.

**Conclusions:**

This study highlights the importance of analyzing molecular interactions and pathways to understand the heterogeneous susceptibility of the host immune response to SARS-CoV-2. We propose new insights into pathogenicity analysis of infections by including genetic risk information as essential factors to predict future complications during and after infection. This approach may assist more precise clinical decisions and accurate treatment plans to reduce COVID-19 complications.

## Background

Immune-mediated inflammatory lung damage is caused by severe COVID-19 infections. Following SARS-CoV-2 infection, host genetic variations contribute to the development of illnesses that require critical care or hospitalization [1, 2]. Genome-wide association studies (GWAS) have been conducted to identify and validate risk genetic variants that significantly impact the severity of COVID-19 among different populations [3, 4]. These studies compared the genomes of patients with severe infections to those of uninfected or mildly affected individuals in order to understand the heterogeneity of the immune responses observed among patients with COVID-19 and discover the underlying disease mechanisms. Although GWAS can be leveraged to determine the associations between genetic variants and the severity of COVID-19, most of the risk variants identified are located in non-coding loci. Some of these studies applied statistical analyses such as Mendelian Randomization (MR) [5] and co-localization [6]. Such approaches help in identifying novel genetic variants in various loci in the host human genome that are associated with the severity of COVID-19 and respiratory failure [6, 7]. Although non-coding variants cannot be directly interpreted, these loci often surround multiple genes. Hence, researchers can infer the effects of these loci on gene expression by integrating multi-omics data [8].

Although previous studies have identified risk variants associated with severe COVID-19, the biological functions and pathways of most identified risk variants and their impacts on the immune system are poorly explained [9, 10]. Such pathways can be interpreted by constructing molecular networks for omics identified as risk factors for severe COVID-19. For instance, various immunological studies concentrated on examining the susceptibility of the immune system response to SARS-Cov-2. Two studies have examined the effects of variants in developing severe COVID-19 by analyzing the function of active proteins of Cytokines response and identifying genetic factors in the interferon circuit during infection [10, 11]. The studies have demonstrated that interferons (IFN-I) play vital roles in the governance of the pathogenesis of COVID-19. The genetic analysis that has been found in the enrichment of (IFN-I) genes and autoantibodies assists doctors in stating and determining the individuals who are at the critical stage of life-threatening COVID-19 [10, 12, 13].Furthermore, functional enrichment analysis can be applied to estimate the molecular function of each risk factor and its contribution to the constructed network. However, there has been no combined analysis of the interactions between all of the proteins in these networks; thus, the interactions or links between these risk factors remain unknown. Hence, mapping genetic risk variants with related diseases and biomarkers is vital to discover and predict possible missing interactions and expand the networks [14].

**Fig. 1:**
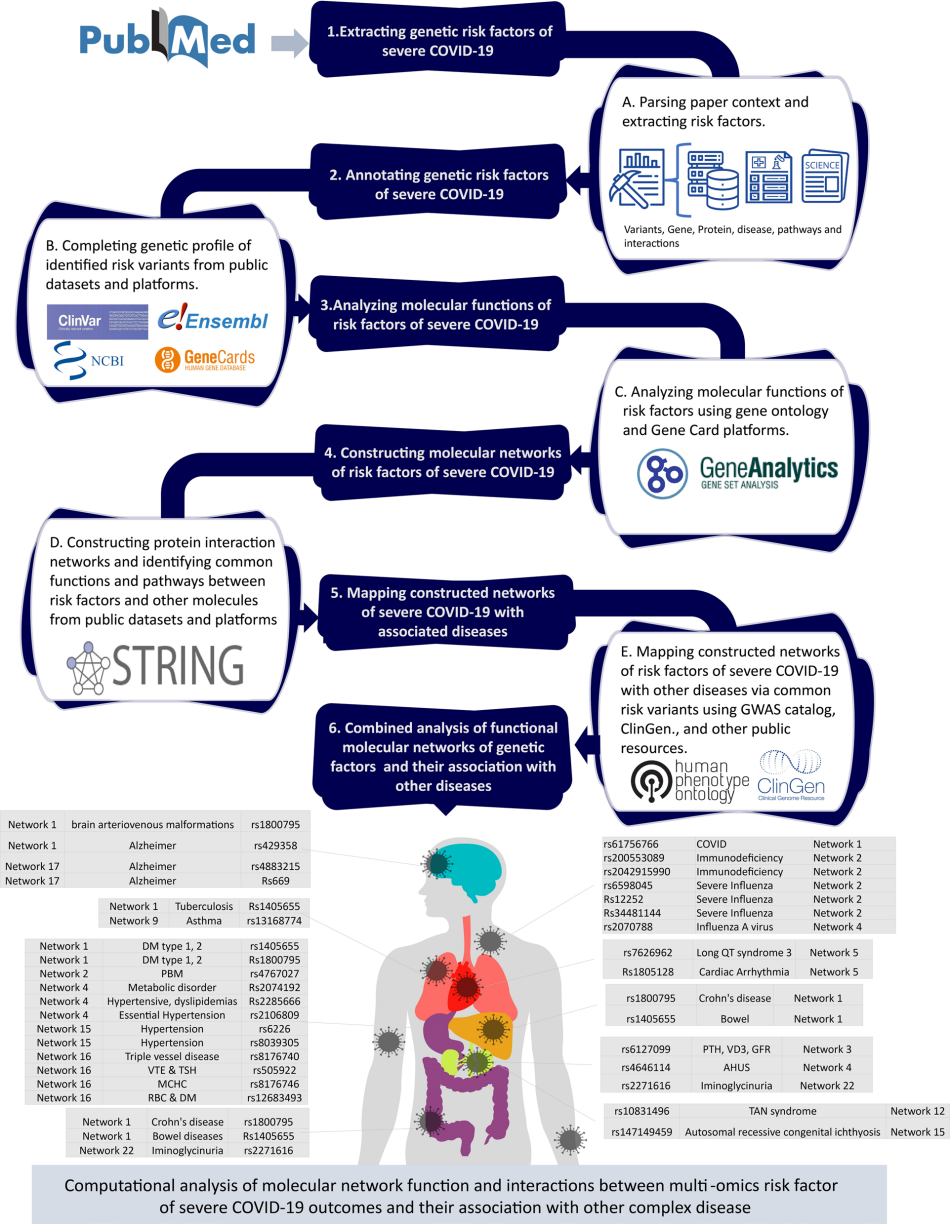
Flow diagram of the study overview. (A) The first step: extracting genetic risk factors of severe COVID-19, by parsing articles and extracting risk factors. such as Variants, Genes, proteins, related diseases, pathways, and interactions. (B) The second step: annotating the genetic risk factors of severe COVID-19 to complete the genetic profiles of identified risk variants from public datasets and platforms. (C) The third step: analyzing molecular functions of risk factors of severe COVID-19 using gene ontology and Gene Card platforms. (D) The fourth step: constructing molecular networks and identifying functions and pathways between risk factors and other molecules from public datasets and platforms. (E) The fifth step: mapping constructed networks of severe COVID-19 with other diseases via shared risk variants using GWAS catalog, ClinGen., and other public resources

Thus, in this paper, we aim to further interpret the biological mechanisms and molecular pathways involved in the pathogenicity of severe COVID-19 by analyzing identified risk variants and inferring the hidden interactions between these molecular networks based on disease mapping. A computational analysis workflow is designed to retrieve genetic risk variants from several biological public resources and available platforms and annotate these risk variants with features to provide deeper insight into each risk variant. We adopted a disease-variant-network mapping approach to perform enrichment analysis and identify the similarity between molecular functions and networks, connect the constructed networks, and infer the invisible links between the constructed protein–protein interaction (PPI) networks. Finally, we map the genetic features and shared biological pathways of the phenotype of the host response to SARS-CoV-2. This analysis identified relevant host genetic factors that are involved in the progression of severe COVID-19 and also related to other diseases, such as metabolic diseases. Thus, this work could help clinicians to identify the hidden genetic factors that influence severe outcomes before symptoms appear and provide more intensive or timely treatment to reduce disease complications. A general overview of the study is visualized in the following flow diagram to illustrate our analysis concept is shown in Fig. 1.

## Results

### Distribution of the chromosomal location of risk variants

**Fig. 2:**
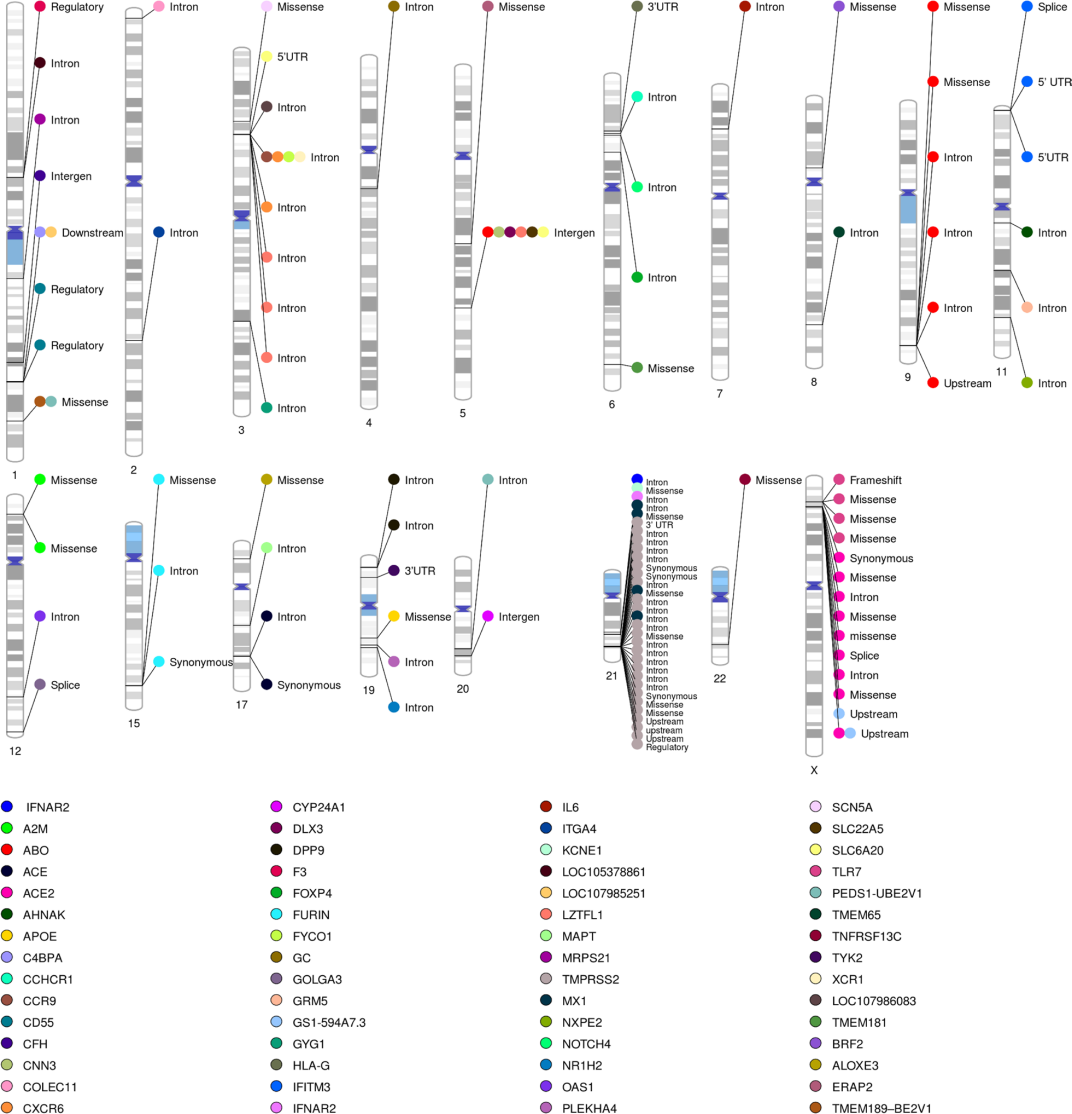
Chromosomal loci and functional consequences of the 109 genetic risk variants. Autosomal loci of the 60 risk genes associated with severe COVID-19: each dot represents a risk variant, and the dots in the same horizontal line represent the same risk variant but in different genes. The colors of the dots represent risk genes

The distribution of the 109 risk variants after filtration on the human chromosomes is presented in Fig. 2. The ideogram shows chromosomes 1-9, 11, 12, 15, 17, and 19-X contain 109 risk variants with different types of variant function such as intergenic, intronic, and transcript variants that are distributed in 60 genes in the host genome. We found two gene clusters that have an impact on severe outcomes of COVID-19. The first gene cluster contains *C4BPA*, *LOC107985251*, *CFH*, and *CD55* located on chromosome 1q31.3-1q.32.2. The loci hold cluster of risk genes are related to immune responses and chemokine and cytokine activity. This cluster contains the regulatory variants rs61821041 and rs61821114, which contribute to downregulation of *CD55*. Furthermore, rs45574833 is associated with atypical hemolytic uremic syndrome, a condition in which thrombi develop in tiny blood arteries in the kidneys. The regulator of complement activation (RCA) contains many tandemly clustered genes with homologous immune system activities, which are downregulated during COVID-19 infection [15]. In addition, the second cluster of genes: *SCN5A*, *LZTFL1*, *SLC6A20*, *FYCO1*, *CCR9*, *CXCR6*, and *XCR1* located on 3p21.31 and 3p22.2 are related to innate immune system activities. For instance, *SCN5A* in human macrophages functions as a pathogen sensor and modulates antiviral responses and defense [16], the cluster is carried by 50% of South Asian and 16% of European populations, and was previously associated with severe COVID-19 and immune dysfunction inherited from Neanderthals [17, 18]. Therefore, our investigation further emphasizes that this cluster is associated with severe COVID-19.

Moreover, the ideogram of the chromosomal locations of identified risk variants associated with severe COVID-19 illustrates that three haplotypes are located on chromosomes 9, 11, and X. Our investigation, we found that these haplotypes influence the immune response and metabolic system. Loci 9q34.3 holds a haplotype that contains six risk variants located in the *ABO* gene. This haplotype interacts with *FUT1-6* and *FUT* and negatively influences the biosynthesis of the components of the blood group systems during COVID-19 infection. Additionally, a second *IFITM3* haplotype on chromosome 11 was previously associated with higher severity of HIV, Dengue, Ebola, and influenza infections. *IFITM3* is an immune effector protein that is essential for both controlling cytokine production and limiting viral replication. The last haplotype shown on the ideogram is located in the *TLR7* gene on chromosome X.

In addition, a group of risk variants located within the 21q22.11-21q22.3 loci influence down-regulation of the activity of the *IFNAR2* and *MX1* genes, which regulate the function of the interferon receptors and B cell-activating factor receptors that are involved in the immune response to viruses.

Furthermore, we found that a group of variants on chromosome 1 (rs1202980, rs60220284, and rs45574833) located in the *C4BPA*, *LOC107985251*, and *TRIM46* genes are related to breast cancer. Additionally, the rs4341 and rs4343 risk variants located in the *AEC* gene on chromosome 17 are related to Alzheimer disease and hypertension. Moreover, rs429358 and rs481778 located in *APOE* and *PLEKHA4* genes on chromosome 19 are related to Alzheimer disease. More explanation of disease-variant mapping is provided in the supplementary materials.

At the level of single polymorphisms related to severe COVID-19, most chromosomes hold risk variants related to the blood and immune system that impact the immune response and increase the severity of COVID-19 during infection. For example, the risk variants found on chromosome 2 are related to the innate immune system, which is the first step of defense and interaction. In addition, some risk variants impact the level of biomarkers in blood. For example, the risk variants on chromosome 1 increase blood pressure during infection, so the biomarker (D-dimer) level increases. Furthermore, some of these variants influence metabolic pathways that affect body systems. For instance, the risk variant on chromosome 3 negatively impacts glucose metabolism pathways. Also, the length of the chromosome, gene size, and overlapping might affect variant distribution by chromosome.

### Statistical analysis on the curated dataset of genetic risk variants of severe COVID-19 outcomes

In an initial analysis, our curated dataset of risk genetic variants of severe COVID-19 outcomes was analyzed with the list of risk variants with their reported effects. The data has 109 risk variants with 16 rare variants with minor allele frequencies (MAF) less than 0.01 and 93 common variants, as reported in the reviewed papers. Most of the reviewed papers reported MAF, *P* value, and other features. However, not all reviewed articles provided the odd ratio (OR) values of variants reported risk variants. Around 50% of the genetic effects of risk variants are not reported in the reviewed articles and need to be calculated. Thus, we re-estimated the additive effect of risk variants by calculating a new OR value for all risk variants using the reported OR and the MAF of each risk variant. Moreover, the association between the MAFs and genetic effects of risk variants of severe COVID-19 is visualized and explained in Additional file 1.

**Fig. 3:**
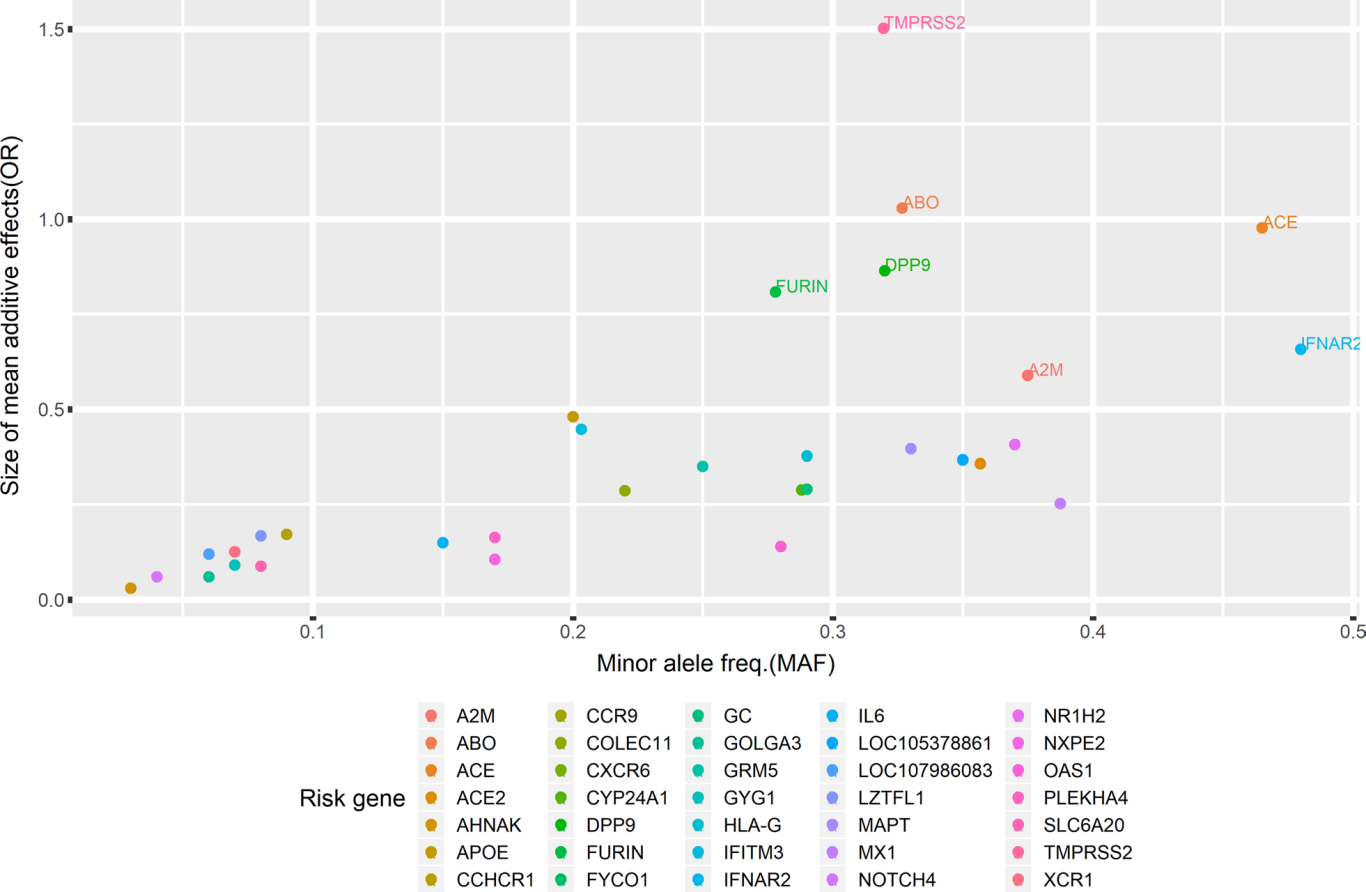
The additive effects of common risk variants on severe COVID-19 outcome per genes. The scatter plot shows the additive effects of common risk variants on severe COVID-19 outcomes per gene. Each point corresponds to the additive effect of the risk gene that has been calculated based on cumulative values of reported ORs and MAF. Each gene hosts at least one reported risk variant

The associations between risk variants and their effects in developing severe outcomes of COVID-19 per gene were established using an additive genetic model estimating the OR of severe COVID-19. The cumulative values of reported OR and MAF of the accumulated risk alleles in a particular gene were calculated and used as a combined effect to estimate the additive effects per gene. Figure 3 shows a scatter plot of the additive effects of risk variants on severe COVID-19 outcomes per gene. Moreover, the statistical method for estimating the genetic effects of severe COVID-19 is explained in Additional file 1.

### Enrichment analysis of genetic risk factors for severe COVID-19

Our curated dataset represents variant profiles that provide descriptive information on risk variants. In our analysis, we named each variant associated with severe COVID-19 in the list as a risk variant and its host gene, as a risk gene causally associated with increased mortality in COVID-19. Furthermore, we named the proteins encoded by risk genes risk proteins. The list of genetic risk variants associated with severe COVID-19 is displayed in Additional file 2: Table S1. Below, we present the enrichment analysis of the genetic risk factors associated with the severity of COVID-19 at different levels: variants, genes, and proteins.

#### Variant level: COVID-19 risk variants and disease association

**Fig. 4:**
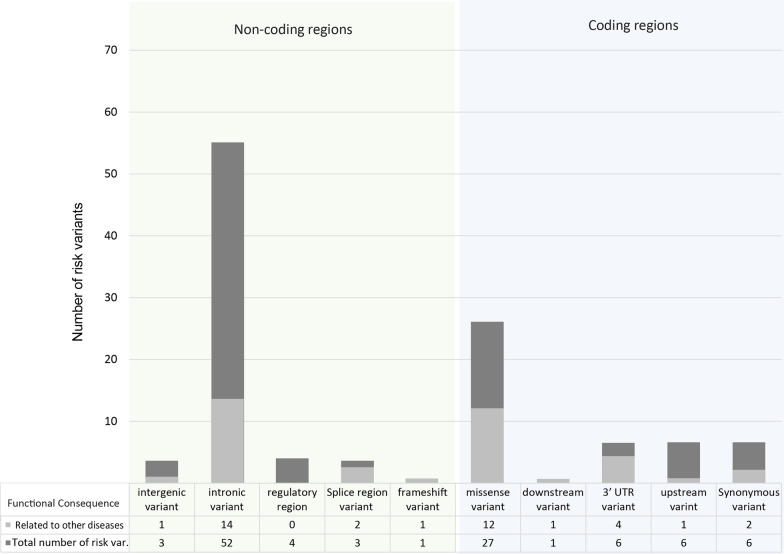
Distribution of functional consequences of the 109 genetic risk variants in the human genome. The height of each bar represents the total number of risk variants in genetic regions, and the light gray color illustrates the number of risk variants associated with other diseases

We found 46 risk variants in coding regions and 63 located in non-coding regions. In non-coding regions, three variants are located in the intergenic region between genes. Fifty-two of the 63 non-coding region variants are intronic variants, and the remainder are transcript variants occurring within intron regions [19, 20]. The distribution of the functional consequences of 109 filtered risk variants in human DNA is shown in Fig. 4.

**Table 1:** List of the risk variants for severe COVID-19 and their associated diseases and biomarkers

Risk variants	Chrom.	Diseases and biomarkers	Related system
rs45574833	1	Atypical Hemolytic Uremic Syndrome (AHUS)	Renal system
rs147149459	3	Hyperglycinuria	Neuron system
rs7626962	3	Heart disease, and Cardiac arrhythmia, Cardiovascular phenotype, and Congenital long QT syndrome	Cardiovascular system
rs61756766	4	High level of white blood cell count procedure biomarker	Immune and hematopoietic systems
rs13168774 ,rs35652899, rs8176746, rs150892504	5	Asthma, and Systemic primary carnitine deficiency (CDSP)	Respiratory and metabolic systems
rs1800795	7	Alzheimer’s disease, Rheumatoid arthritis, KAPOSI Sarcoma, Diabetes mellitus type 1 and 2	Neuron and metabolic systems
rs495828	9	Three-vessel disease (3VD)	Cardiovascular system
rs505922	9	Diabetes mellitus Non-insulin-dependent, and high level of red blood cell count	Metabolic system
rs12683493	9	Mean Corpuscular Hemoglobin Concentration (MCHC)	Hematopoietic system
rs657152	9	Venous thromboembolism, and low level of thyroid stimulating hormone	Hematopoietic system
rs6127099	9	Von Willebrand disease (VWD)	Hematopoietic system
rs12252	11	Severe influenza, hypercholesterolemia, dengue fever, pneumonia, hepatoma, HIV-1 infection	Immune system
rs34481144, rs6598045	11	Severe influenza	Immune system
rs143359233	11	Suntan	Integumentary systems
rs4883215	12	Alzheimer’s disease	Neuron system
rs669	12	Alzheimer’s disease, and Alpha-2-Macroglobulin polymorphism	Neuron system
rs4767027	12	Related to level of blood protein biomarker	Hematopoietic system
rs8039305, rs769208985	15	Hypertension	Circulatory systems
rs72711165	17	Ichthyosis and Lamellar ichthyosis	Integumentary systems
rs4343	17	Alzheimer’s disease, high serum albumin level, Myocardial infarction, and renal tubular dysgenesis of genetic origin	Neuron and metabolic systems
rs4341	17	Diabetes and hypertension, renal tubular dysgenesis of genetic origin	Metabolic and circulatory systems
rs429358	19	Alzheimer disease, hyperlipoproteinemia	Neuron system
rs1405655	19	Diabetes mellitus non-insulin-dependent, Inflammatory bowel diseases, and Tuberculosis (TB)	Immune and metabolic systems
rs2109069	19	Idiopathic pulmonary fibrosis	Respiratory system
rs12610495	19	Lung Diseases Interstitial	Respiratory system
rs74956615	19	Rheumatoid arthritis, Cholangitis sclerosing, Ulcerative colitis, Crohn disease, Psoriasis, and Ankylosing spondylitis	Metabolic and integumentary systems
rs138763430	20	Glomerular Filtration Rate, Vitamin D3 , Parathyroid hormone biomarkers	Hematopoietic and metabolic systems
rs2070788	21	Cardiac arrhythmia, Atrial fibrillation, Long QT syndrome, Cardiovascular phenotype, Jervell and Lange-Nielsen syndrome 2	Cardiovascular system
rs114363287	21	Severe influenza due to influenza A virus subtype H7N9	Immune system
rs11702475	21	Prostate carcinoma	Reproductive system
rs11385942	22	Immunodeficiency	Immune system
rs200553089, rs2042915990	X	Immunodeficiency	Immune system
rs2074192	X	Cardiovascular diseases, Essential hypertension	Cardiovascular and circulatory systems
rs2285666	X	Diabetes mellitus non-insulin-dependent, Hypertensive disease, Dyslipidemias, Orthostatic hypertension	Metabolic and circulatory systems
rs2106809	X	Essential Hypertension	Circulatory systems

Table 1 displays the variant-disease associations for 41 risk variants. Seven risk variants are related to metabolic disorders such as diabetes mellitus, hyperglycemia, and blood protein levels. Three of the intron variants have an impact on *ABO* protein synthesis, which influences the red cell count and is associated with metabolic diseases such as diabetes and venous thromboembolism (VTE) [21, 22]. The rs12683493 risk variant changes glycosylation and causes von Willebrand disease [22, 23].

Moreover, four risk variants are linked with cardiovascular disorders such as cardiac arrhythmia, long QT syndrome, and triple vessel diseases. Three risk variants are associated with immune dysfunction; for example, white blood cell disorders, complete blood count disorders, and rheumatoid arthritis. Three risk variants are mapped to gastrointestinal diseases, such as Crohn’s disease and inflammatory bowel disease. Four risk variants are associated with cancer such as prostate cancer and Kaposi’s sarcoma. Another five risk variants are related to severe symptoms in infectious diseases namely, tuberculosis and severe influenza. One variant is related to respiratory disorders and mapped to severe asthma.

#### Gene level: COVID-19 risk genes and disease association

**Fig. 5:**
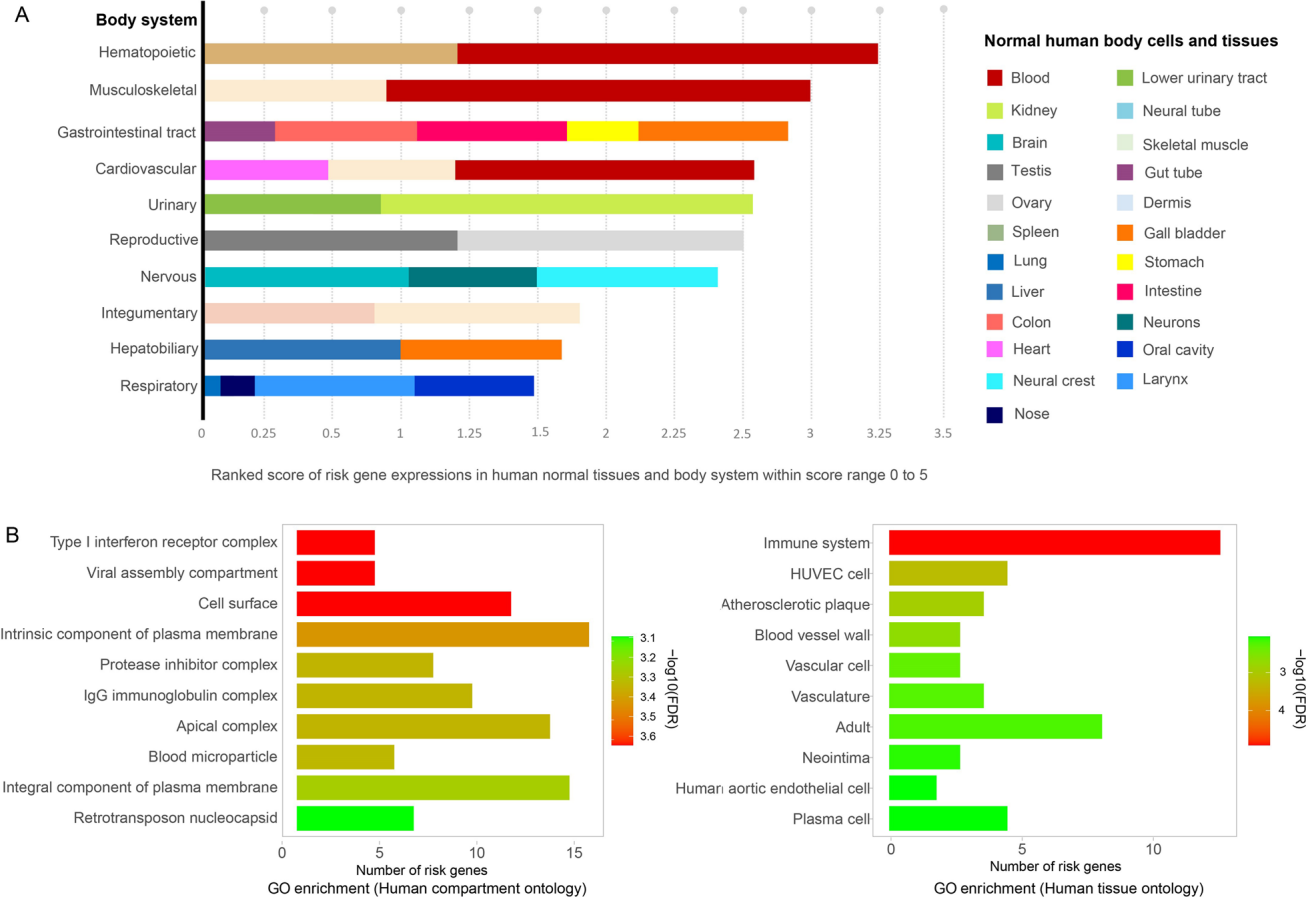
Risk gene set enrichment analysis of the 60 risk genes related to severe COVID-19.** A** The top ten ranked gene expression scores in human normal tissues and systems: the vertical axes represent the top ten systems based on ranked scores. Each bar represents a system, and the slots inside the bar represent the percentage of the risk genes expressed in various human tissues or cells based on the ranked gene expression scores. The horizontal axes represent the ranked score from 0 to 5.** B** The top ten human compartments and tissues with the highest numbers of enriched risk genes based on gene ontology analysis

The results of gene set analysis of the COVID-19 risk genes are shown in Fig. 5. The gene expression of the top ten ranked systems in normal tissues and cells derived from our enrichment analysis of the risk gene set is shown in the bar chart in Fig. 5A. Most of these risk genes are highly expressed in blood cells in the hematopoietic system, with a ranked score of 3.5 related to the immune response and viral interaction. The second system is the musculoskeletal system with a ranked score of 3, with some of the risk variants involved in blood circulation. The other three related systems are the renal, reproductive, and neuro systems and are approximately similar with ranked scores of 2.5. The risk genes for the respiratory system and the lungs only have a ranked score of 1.5.

Screening the list of risk genes can help to comprehend the organs and systems affected in severe COVID-19. However, the presence of gene expression does not necessarily mean that the gene is connected functionally to a network related to its expressed system or tissue. For example, three genes out of 60 risk genes are RNA-encoding genes affiliated with the ncRNA class *LOC105378861*, *LOC107986083*, and *LOC107985251*. The phenotype of *LOC105378861* is related to the levels of tissue factor activity in blood and increased D-dimer levels in patients with COVID-19 [24]. However, no information is available about *LOC107986083* and *LOC107985251*.

Figure 5B illustrates the number of risk genes that are enriched in human compartments and tissues. Mainly, most risk genes are enriched in the immune system and are located on the cell surface and related to the receptor of type I interferon or viral assembly compartments.

**Fig. 6:**
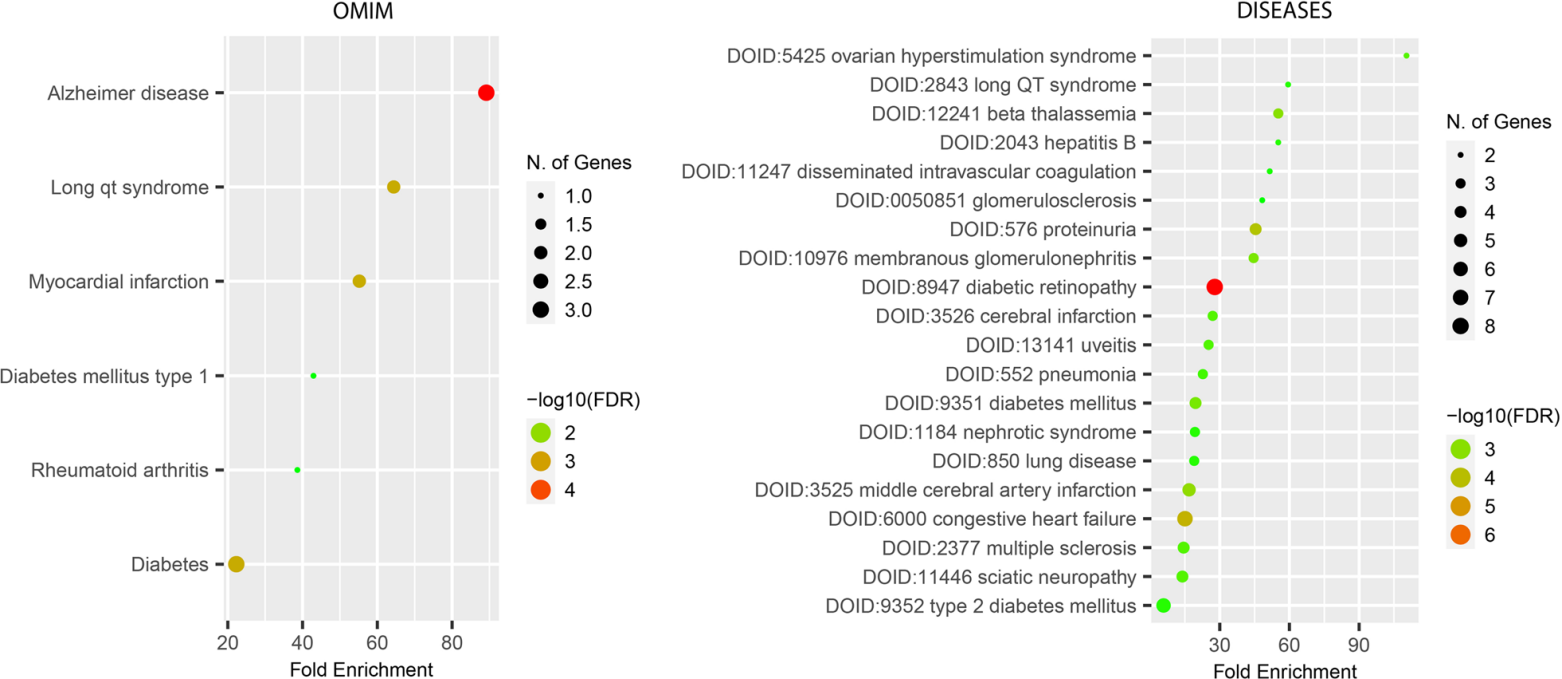
Gene set and disease analysis of the 60 risk genes related to the severity of COVID-19 outcomes. The top diseases were mapped to the risk genes based on the OMIM and Alliance-DISEASES databases. The circle size represents the number of genes associated with the disease, and the range of colors (high-significant level: red, low-significant level: green) represents the FDR scores for the disease associations

The results of the risk gene analysis with regard to disease associations are illustrated in Fig. 6. The top most-enriched risk genes mapped to disease based on the Mendelian Inheritance in Man (OMIM) database [25]. Inherited Alzheimer disease was the top disease that mapped to the risk genes, followed by long QT syndrome, myocardial infections, metabolic diseases, and lung dysfunction.

#### Protein level: COVID-19 risk proteins and molecular functional analysis

We found 56 proteins involved in the development of severe COVID-19. We applied GO enrichment analysis of the risk proteins derived from our dataset after eliminating three genes that did not encode proteins. This analysis showed 24 proteins are involved in the immune system.

**Fig. 7:**
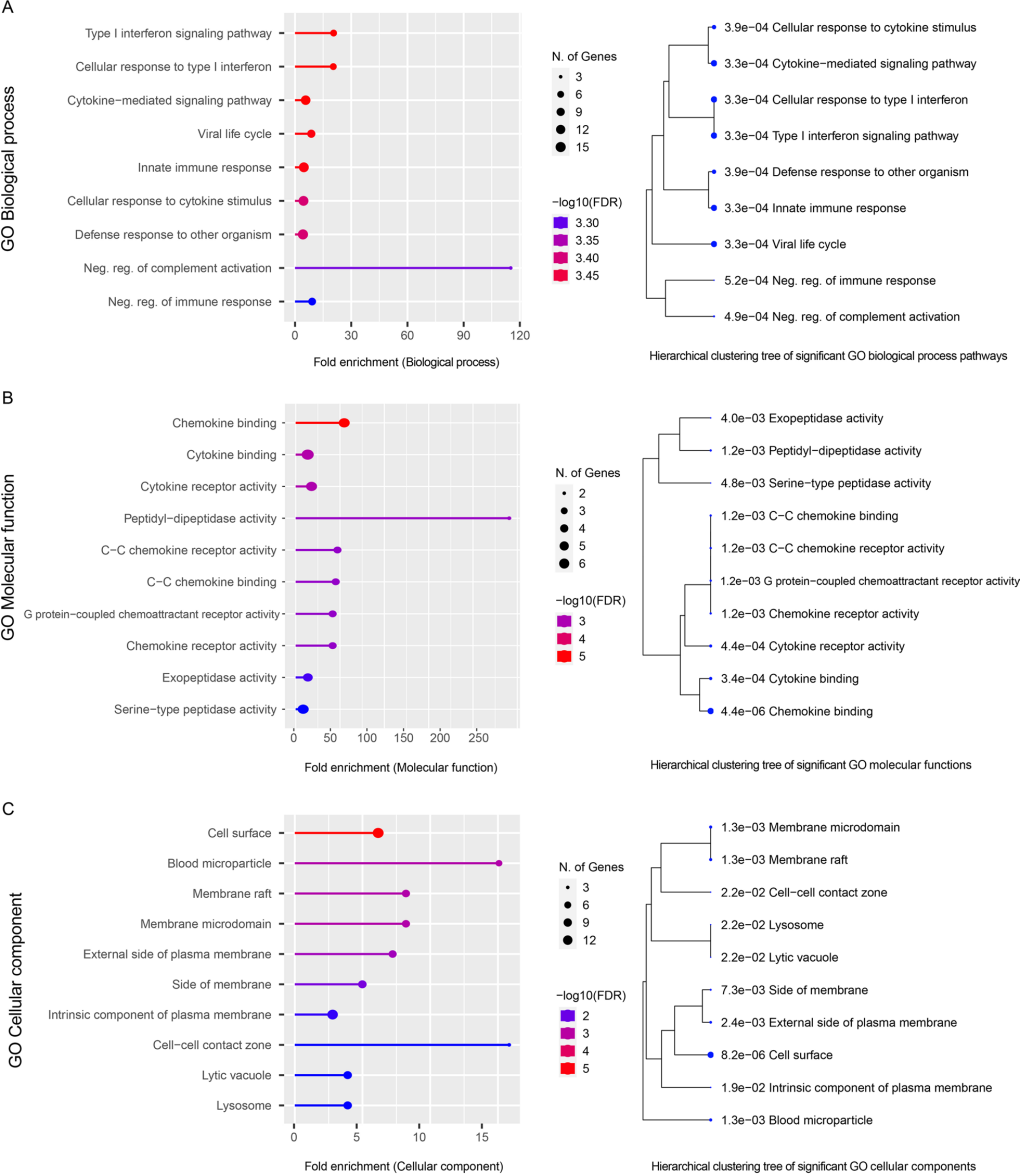
GO enrichment analyses of the 56 risk proteins related to severe COVID-19 outcomes.** A** The top ten significant biological processes and hierarchical correlation clustering trees of the biological processes enriched with the risk proteins.** B** The top ten significant molecular functions and hierarchical correlation clustering trees of the molecular functions enriched with the risk proteins.** C** The top ten significant cellular components and hierarchical correlation clustering trees of the cellular components enriched with the risk proteins

Figure 7A shows the risk proteins are mainly enriched in the following biological processes: negative regulation of complement activation, response to interferon-beta, type I interferon signaling pathways, and cellular response to type I interferon. In terms of molecular function, Fig. 7B illustrates the risk proteins are highly enriched in peptidyl-dipeptidase activity, and chemokine binding with high false discovery rate (FDR) scores; these activities are involved in the immune system response. In addition, the results of the enrichment analysis of risk proteins in terms of cellular components are shown in Fig. 7C. The risk proteins are enriched in blood microparticles. Furthermore, the clustering trees summarize the correlation between the significant molecular pathways and functions. The pathways with many shared risk proteins cluster together in one branch. More significant *P* values are indicated by larger circles.

**Table 2:** Main host biological processes associated with the 56 risk proteins that contribute to the development of severe COVID-19

Number of risk protein	High level GO biological process category	Risk protein list
24	Immune system process	CFH, OAS1, TYK2, ITGA4, COLEC11, C4BPA, C3, APOE, NR1H2, IL6, FURIN, IFITM3, MX1, IFNAR2, ACE, TNFRSF13C, GYG1, CXCR6, A2M, CD55, TLR7, NOTCH4, HLA-G, MAPT
22	Response to stress	*CFH, OAS1, BRF2, TYK2, F3, COLEC11, C4BPA, C3, APOE, ACE2, NR1H2, IL6, FURIN, IFITM3, MX1, IFNAR2, CXCR6, A2M, CD55, TLR7, HLA-G, MAPT*
22	Response to external stimulus	*CFH, CYP24A1, OAS1, TYK2, ITGA4, F3, COLEC11, C4BPA, C3, APOE, ACE2, NR1H2, IL6, FURIN, IFITM3, MX1, IFNAR2, CXCR6, A2M, CD55, TLR7, HLA-G*
21	Immune response	*CFH, OAS1, TYK2, ITGA4, COLEC11, C4BPA, C3, APOE, NR1H2, IL6, FURIN, IFITM3, MX1, IFNAR2, TNFRSF13C, GYG1, CXCR6, A2M, CD55, TLR7, HLA-G*
21	Regulation of response to stimulus	*CFH, OAS1, PLEKHA4, ITGA4, F3, COLEC11, C4BPA, C3, APOE, ACE2, NR1H2, IL6, FURIN, IFNAR2, A2M, SCN5A, CD55, TLR7, NOTCH4, HLA-G, MAPT*
18	Regulation of signaling	*OAS1, PLEKHA4, F3, C3, APOE, ACE2, NR1H2, IL6, FURIN, IFNAR2, TMEM65, GRM5, A2M, CD55, TLR7, NOTCH4, HLA-G, MAPT*
18	Biological process involved in interspecies interaction between organisms	*CFH, OAS1, TYK2, COLEC11, C4BPA, C3, APOE, ACE2, NR1H2, IL6, IFITM3, MX1, IFNAR2, A2M TMPRSS2, CD55, TLR7, HLA-G*
18	Regulation of multicellular organismal process	*OAS1, F3, C3, APOE, ACE2, NR1H2, IL6, FURIN, ACE, TMEM65, A2M, KCNE1, SCN5A, CD55, TLR7, NOTCH4, HLA-G, MAPT*
16	Regulation of immune system process	*CFH, OAS1, ITGA4, COLEC11, C4BPA, C3, APOE, NR1H2, IL6, FURIN, IFNAR2, TNFRSF13C, A2M, CD55, TLR7, HLA-G*
16	Response to biotic stimulus	*CFH, OAS1, TYK2, COLEC11, C4BPA, C3, APOE, NR1H2, IL6, IFITM3, MX1, IFNAR2, A2M ,CD55, TLR7, HLA-G*
16	Response to other organism	*CFH, OAS1, TYK2, COLEC11, C4BPA, C3, APOE, NR1H2, IL6, IFITM3, MX1, IFNAR2, A2M, CD55, TLR7, HLA-G*
16	Regulation of biological quality	*OAS1, F3, APOE, ACE2, NR1H2, IL6, FURIN, MX1, ACE, GRM5, CXCR6, A2M, KCNE1, SCN5A, CD55, MAPT*
15	Regulation of localization	*ITGA4, F3, AHNAK, C3, APOE, ACE2, NR1H2, IL6, FURIN, ACE, LZTFL1, GRM5, KCNE1, SCN5A, MAPT*
15	Regulation of molecular function	*OAS1, ITGA4, F3, AHNAK, C3, APOE, ACE2, NR1H2, IL6, FURIN, ACE, GRM5, A2M, KCNE1, MAPT*
12	Cellular localization	*ITGA4, C3, APOE, MX1, GYG1, LZTFL1, A2M , KCNE1, SCN5A, CD55, CCHCR1, MAPT*
11	Anatomical structure morphogenesis	*PLEKHA4, ITGA4, F3, C3, APOE, IL6, FURIN, SCN5A, NOTCH4, HLA-G, MAPT*
11	Macromolecule localization	*ITGA4, C3, APOE, NR1H2, IL6, FURIN, ACE, LZTFL1, KCNE1, CCHCR1, MAPT*
10	Immune effector process	*CFH, COLEC11, C4BPA, C3, IL6, ACE, GYG1, A2M, CD55, HLA-G*
10	System process	*APOE, ACE2, NR1H2, ACE, SLC6A20, TMEM65, GRM5, KCNE1, SCN5A, MAPT*
10	Cell population proliferation	*ITGA4, F3, APOE, ACE2, IL6, ACE, TNFRSF13C, SCN5A, CD55, HLA-G*
10	Cellular component biogenesis	*OAS1, BRF2, APOE, ACE2, NR1H2, MX1, ACE, LZTFL1, HLA-G, MAPT*
9	Regulation of developmental process	*PLEKHA4, F3, C3, APOE, NR1H2, IL6, NOTCH4, HLA-G, MAPT*
8	Leukocyte activation	*ITGA4, C3, IL6, TNFRSF13C, GYG1, CD55, HLA-G, MAPT*
7	Biological adhesion	*ITGA4, ACE2, IL6, TNFRSF13C, CD55, NOTCH4, HLA-G*
7	Catabolic process	*CYP24A1, C4BPA, APOE, IL6, FURIN, ACE, MAPT*
7	Response to endogenous stimulus	*ITGA4, APOE, IL6, FURIN, GRM5, KCNE1, MAPT*
7	Anatomical structure formation involved in morphogenesis	*ITGA4, F3, C3, IL6, FURIN, NOTCH4, HLA-G*
6	Locomotion	*ITGA4, F3, APOE, IL6, ACE, CXCR6*
6	Activation of immune response	*CFH, COLEC11, C4BPA, C3, A2M, CD55*
6	Cell adhesion	*ITGA4, IL6, TNFRSF13C, CD55, NOTCH4, HLA-G*
6	Regulation of cell adhesion	*ITGA4, IL6, TNFRSF13C, CD55, NOTCH4, HLA-G*
6	Cell motility	*ITGA4, F3, APOE, IL6, ACE, CXCR6*
6	Localization of cell	*ITGA4, F3, APOE, IL6, ACE, CXCR6*
5	Immune system development	*ITGA4, IL6, ACE, NOTCH4, HLA-G*
5	Regulation of locomotion	*ITGA4, F3, APOE, IL6, ACE*
5	Regulation of cellular component biogenesis	*APOE, ACE2, NR1H2, ACE, MAPT*
4	Cell killing	*CFH, C3, CD55, HLA-G*
4	Behavior	*APOE, ACE2, GRM5, MAPT*
4	Maintenance of location	*C3, APOE, NR1H2, IL6*
3	Reproduction	*GOLGA3, C3, ACE*
3	Reproductive process	*GOLGA3, C3, ACE*
3	Growth	*ITGA4, APOE, MAPT*
3	Production of molecular mediator of immune response	*IL6, CD55, HLA-G*
3	Developmental process involved in reproduction	*GOLGA3, C3, ACE*
3	Cell growth	*ITGA4, APOE, MAPT*
3	Regulation of cell killing	*CFH, CD55, HLA-G*
3	Taxis	*F3, IL6, CXCR6*
3	Hormone metabolic process	*ACE2, FURIN, ACE*
3	Developmental growth	*ITGA4, APOE, MAPT*
3	Complement-dependent cytotoxicity	*CFH, C3, CD55*
3	Regulation of hemostasis	*F3, APOE, A2M*
2	Multi-organism process	*GOLGA3, ACE*
2	Locomotory behavior	*APOE, GRM5*
2	Response to abiotic stimulus	*TLR7, MAPT*
2	Antigen processing and presentation	*ACE, HLA-G*
2	Sexual reproduction	*GOLGA3, ACE*
2	Multicellular organism reproduction	*GOLGA3, ACE*
2	Regulation of growth	*APOE, MAPT*
2	Multi-organism reproductive process	*GOLGA3, ACE*
2	Multicellular organismal reproductive process	*GOLGA3, ACE*
2	Leukocyte migration	*ITGA4, IL6*
2	Protein activation cascade	*F3, A2M*
2	Regulation of plasma lipoprotein particle levels	*APOE, NR1H2*

Table 2 displays the groups of risk proteins involved in biological process pathways related to immune and metabolic activities that contribute to the development of severe COVID-19 symptoms.

### Summary of the enrichment analysis of risk factors for severe COVID-19

In summary, after applying a comprehensive enrichment analysis of our curated dataset of 109 risk variants associated with severe COVID-19 at different levels (variants, genes, and proteins), we mapped the genetic factors to related diseases to infer the relationships between severe COVID-19 and future complications. Based on the chromosomal distribution and risk variant-disease mapping, we identified three clusters of genes related to immune, hematopoietic, and metabolic dysfunction. Furthermore, three haplotypes contribute to hematopoietic and immune system complications. We found a haplotype of contiguous variants that contribute to regulatory functions or are associated with other diseases that cause severe COVID-19 symptoms. Six risk variants are located within contiguous loci in *ABO* involved in glycosylation metabolism. In addition, it is notable that the *TMRSS2* and *ACE2* receptors contain a massive number of contiguous variants that may increase the susceptibility of host cells to viral infection. Numerous groups of variants on chromosomes 21 and X influence the antigenic response of SARS-CoV-2 variants. Moreover, polymorphisms have an influence on host immune recognition and the susceptibility and intensity of the immune response to the SARS-CoV-2 virus [7, 24, 26–29]. Hence, we applied enrichment analysis on the set of host risk variants to evaluate gene differentiation and discover biomarkers for tissues and cells from the set. The results show that most genes are expressed in both the hematopoietic and immune systems. Furthermore, the molecular functions and biological processes of the list of proteins encoded by risk genes illustrate these proteins are involved in immune responses and metabolic activities.

### Molecular network construction and integration

We eliminated four genes that did not encode proteins. Then, we constructed protein–protein interaction networks for 56 risk proteins that contain a total of 939 interacting proteins, including 48 risk proteins with 939 interactions, and seven orphan proteins that did not interact with any protein. We integrated all constructed networks for the risk proteins and obtained 24 connected PPI networks and seven orphan proteins. In addition, Table 3 displays the main systems and host tissues involved in the constructed networks and affected by the risk factors for severe COVID-19. More details of the PPI networks and related functions are displayed in Additional file 3.

**Table 3:** Summary of the 24 molecular networks constructed using the genetic risk factors for severe COVID-19

Network ID	Risk variants	Risk protein	Host protein	PPI	Main related system	Main related tissues and cells
1	24	16	117	511	Immune system	Blood cells
2	7	5	37	75	Innate immune system	Liver and digestive gland
3	14	3	15	27	Renin-angiotensin and metabolomic systems	Kidney and viscus tissue
4	1	3	11	27	Vascular system	Heart and viscus tissue
5	1	1	11	13	Cardiovascular system	Cardiac muscle fiber and heart tissue
6	1	1	11	55	Ribosomal protein	Pituitary gland tumor cell
7	4	1	11	33	Metabolic system	Kidney, heart, liver and adipose
8	1	1	11	19	Hematopoietic system	Natural killer cell and blood cells
9	1	1	11	28	Metabolic system	Muscle tissue
10	1	1	11	19	Immune system	Blood and natural killer cells
11	1	1	11	38	Central nervous system	Cerebral cortex and brain tissue
12	3	1	11	12	Endomembrane system	Ovary and testis tissue
13	7	1	8	24	Metabolic system	Esophagus tissue
14	2	2	8	11	Metabolic system	Liver
15	1	1	7	14	Innate Immune system	Low tissue specificity
16	7	1	6	13	Immune system	Lymphoid tissue in B-cells
17	1	1	5	7	Urinary and cardiovascular systems	Kidney and heart tissue
18	1	1	5	6	Cardiovascular system	Heart and skeletal muscle
19	1	1	3	2	Immune system	Blood cells
20	25	1	2	1	Immune system	VCaP cell
21	2	1	2	1	Immune system	Kidney and small intestine
22	1	1	2	1	Immune and hematopoietic systems	Blood cells
23	1	1	2	1	Immune and hematopoietic systems	Blood cells
24	1	1	2	1	Immune and hematopoietic systems	Blood cells

Based on the functional analysis above, most networks are related to the blood and immune systems. However, there is no apparent interaction that connects the proteins in these networks, which points to a missing interaction or link. Thus, to highlight such missing links, we adopted another approach to correlate the constructed networks with common diseases that are related to the risk variants hosted in these networks.

**Fig. 8:**
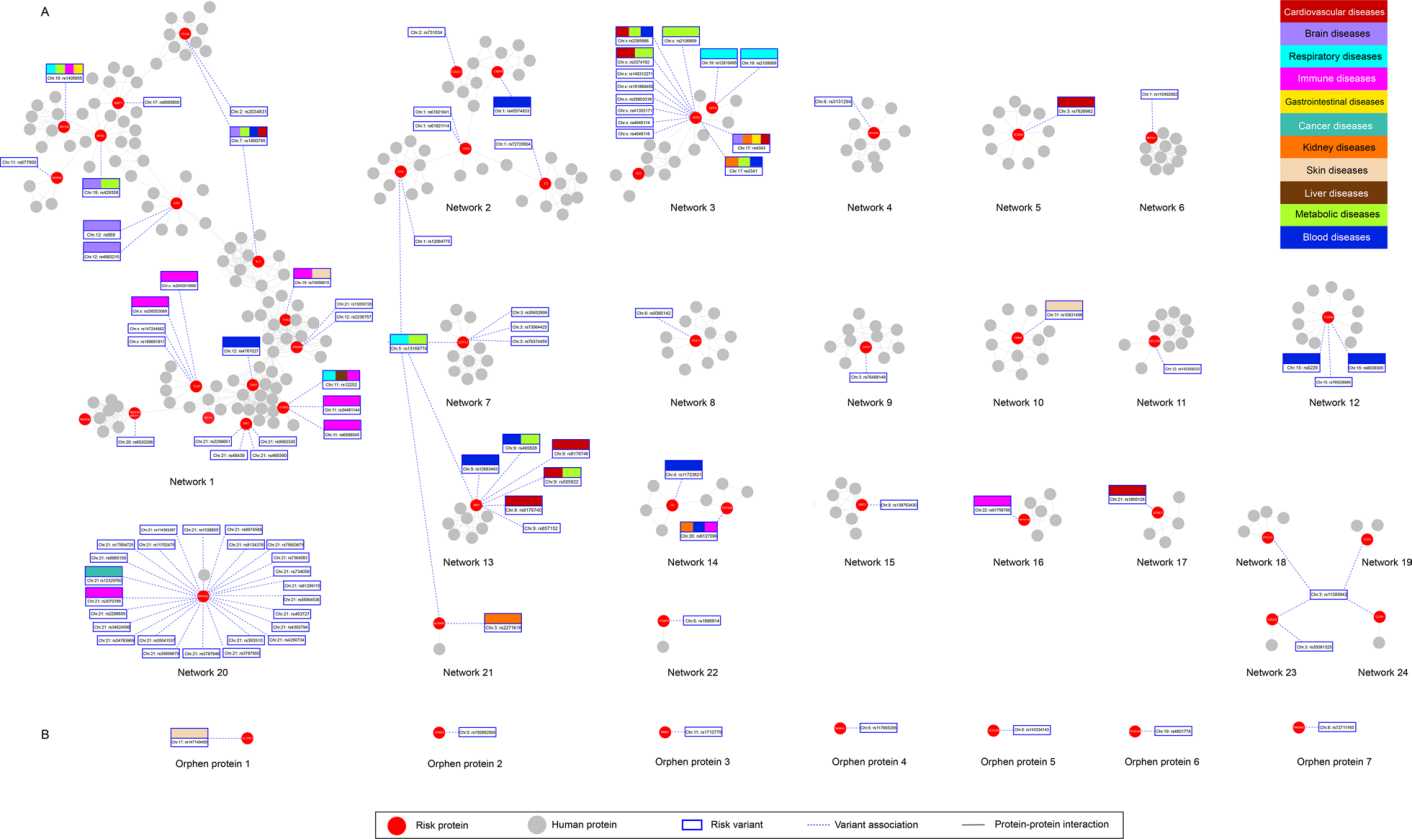
Molecular networks of the 56 risk proteins mapped with the 109 risk variant-disease associations. The red nodes represent COVID-19 risk proteins. The gray nodes represent human proteins that interact with risk proteins.** A** Twenty-four connected networks. Each connected network has at least one interaction with another protein. Networks 1 to 3 contain more than one risk protein, and Networks 4 to 22 are isolated networks has one risk protein.** B** Seven orphan risk proteins did not have any protein interaction with other human proteins. The dashed lines link risk variants with linked networks. The different colors of squares illustrate the disease mapping based on the risk variant-disease mapping and the similarity of the molecular functions between the constructed networks

Figure 8 illustrates the 24 PPI constructed based on the results of the risk variant-disease mapping in order to indirectly infer the common molecular functions between the constructed PPI networks. The variant-disease mapping is listed in Additional file 2: Table S2.

According to our investigation of the 24 constructed PPI networks, we infer that the molecular functions of the risk protein interactions are involved in three main host systems: the immune, metabolic, and cardiovascular systems.

In terms of risk variant mapping, Networks 2, 7, and 21 have a common risk variant rs13168774 located on chromosome 5 and associated with respiratory inflammation and metabolic disorders. Moreover, Networks 18, 19, 23, and 24 have a common risk variant rs11385942. These networks have common molecular functions related to immune responses. With reference to the similarity of biological processes and molecular functions between networks, Networks 1, 2, 9, 10, 15, 16, and 19-24 are related to the immune and hematopoietic systems. Networks 3, 7, 13, 14, and 17 are related to the metabolic system. Networks 4, 5, 17, and 18 are related to the cardiovascular system. Networks 3 and 17 are related to the urinary system. Network 11 is related to the nervous system. Network 12 is related to the endomembrane system.

Based on the evidence gleaned from the molecular function analysis of the constructed networks and risk variant-disease mapping, we inferred the hidden PPIs between unconnected networks. The molecular function analysis revealed several hidden pathways. For example, we found unconnected Networks 1-3, 6-10, and 12-24 have similar molecular functions and biological processes related to the immune system. In addition, we inferred unconnected Networks 1-5, 13, 14, and 17-16 share common pathways related to the cardiovascular system and diseases. Moreover, Networks 1, 3, and 11 have common functions and diseases related to the neuron system. Furthermore, based on the disease mapping similarity, we derived that unconnected Networks 1-3, 15-17, and 21 have pathways related to the renal system. Thus, we infer hidden interactions could connect these networks.

### Molecular pathways of constructed networks

**Fig. 9:**
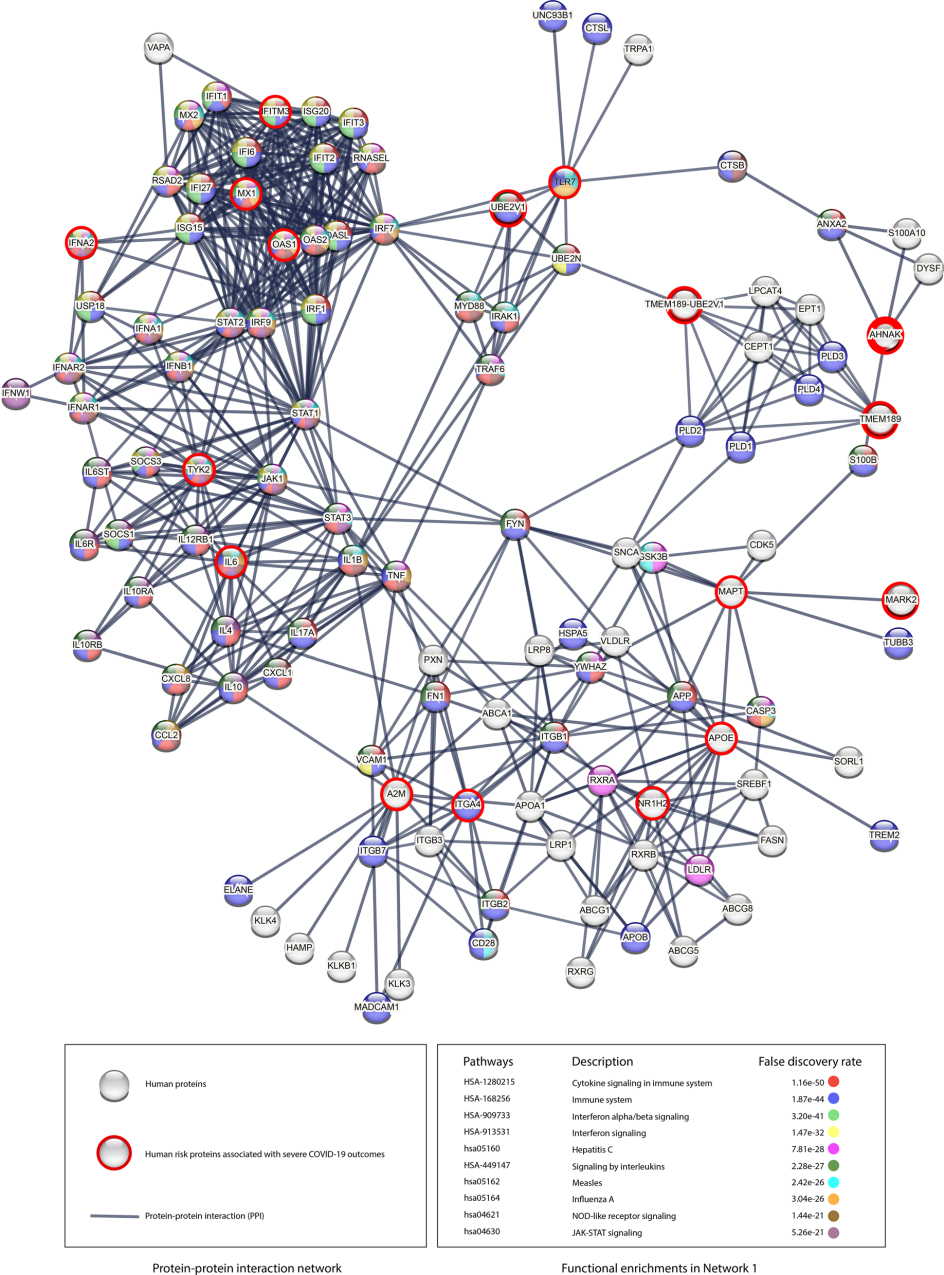
Molecular pathways between the 16 genetic risk proteins of severe COVID-19 in Network 1. The top ten significant molecular pathways between the genetic risk proteins and other host proteins are mainly connected to the host immune system. Network 1 is the largest network and contains the highest number of risk proteins compared to other networks

Out of the 24 constructed networks, we analyzed the pathways in Network 1, the largest network that has the highest number of risk proteins (n = 16) and pathways related to the host immune system and contains 511 interactions. Based on the top ten functional pathways of Network 1, we found that eight of the ten molecular pathways in Network 1 are related to cytokine signaling and responses in the immune system and SARS-CoV-2 responses in the innate immune system. The remaining three pathways are related to Influenza A, measles, and Hepatitis C infections. Figure 9 shows the molecular pathways for the risk factors from Network 1; this network is related to the immune system, which confirms that the majority of pathways in Network 1 are related to immune responses. Moreover, the functional pathways in Network 2 contain five risk proteins and 75 interactions that are mainly related to the innate immune system. We found that Network 2 has eight significant pathways with *P* values higher than 2.6e−13 related to the complement and coagulation cascade pathways. The complement system is a central component of the innate immune system.

**Fig. 10:**
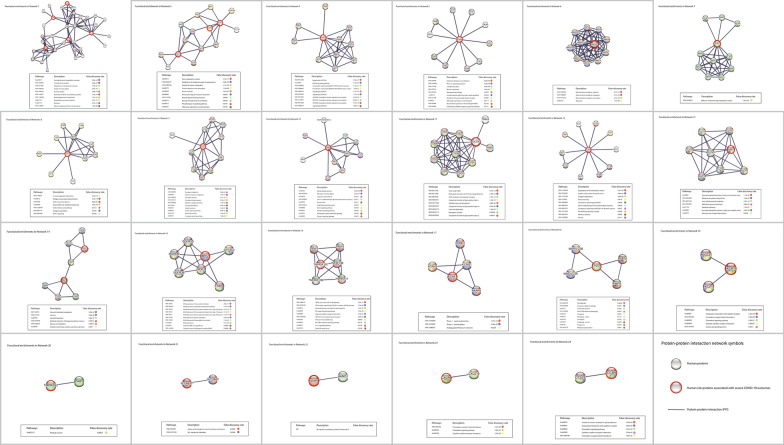
Overview of the molecular pathways of the risk proteins in the constructed networks related to the host metabolic, cardiovascular, and other systems. The figure demonstrates the significant pathways related to severe COVID-19 outcomes. More details of the remaining constructed networks are provided in Additional file 4

However, some constructed networks are not related to the immune system. For instance, Network 3 contains three risk proteins and 27 interactions and is related to the metabolic and renin-angiotensin systems, which play an essential role in the regulatory functions and processes of renal, cardiac, and vascular metabolism and physiology. We found that Network 3 has five significant pathways with *P* values < 5e−08 related to peptide hormone metabolism and protein digestion and absorption, which are involved in metabolic processes and systems. In addition, Network 4 contains three risk proteins and 27 interactions related to the cardiovascular system. We found that all top ten pathways in Network 4 are involved NOTCH signaling and the intracellular domain of NOTCH regulates transcription of genes related to cardiac development. Figure 10 shows the molecular pathways of the remaining constructed Networks 2-24. More details of the molecular pathways between the proteins in these networks and pathway analysis of the remaining constructed networks are demonstrated in Additional files 3 and 4.

### Host genetic risk factors and the SARS-CoV-2 pathway

After applying molecular function enrichment analysis and disease mapping to risk factors related to severe COVID-19, we found that the majority of pathways are related to the immune system, which suggests that the risk factors associated with COVID-19 are mostly present in proteins involved in the immune system. A minority of networks that have three or more risk proteins, such as Networks 3 and 4, are related to the metabolic and cardiovascular systems. This evidence indicates that the genetic risk factors associated with severe COVID-19 are involved in different host systems that cause multi-organ dysfunctions.

**Fig. 11:**
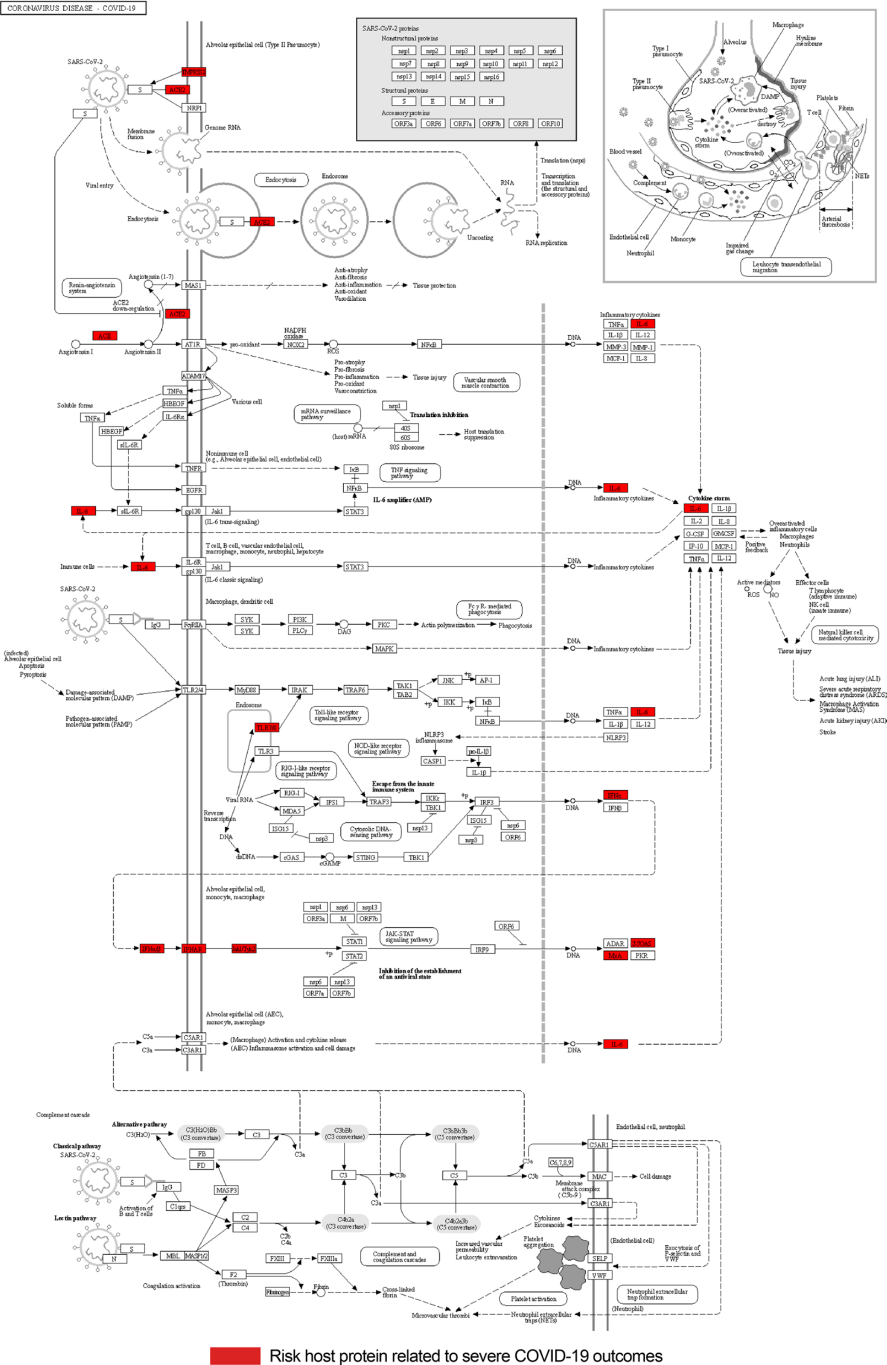
Host-SARS-CoV-2 pathogen interaction pathway in COVID-19. The location of the 56 genetic risk proteins in the main molecular pathway involved in the host response to SARS-CoV-2 derived from KEEG database. The proteins involved in this pathway are components of different constructed molecular networks, such as Network 1, 3, and 20

Figure 11 shows the location of the risk proteins in the main host-virus pathway, which contains ten risk proteins from different constructed molecular networks. For instance, *IL-6*, *TLR7*, *OAS*, and *IFNAR* from Network 1 and *TMPRSS2* from Network 20 are mainly related to immune system and cytokine and interferon signaling. In addition, *ACE* and *ACE2* from Network 3 are related to the metabolic system and processes.

## Discussion

The limited understanding of biological mechanisms and the impact of host genetic risk factors on severe COVID-19 has led researchers to identify genetic risk variants and analyze their influence on the development of severe symptoms. Our study is analogous to the molecular network analysis studies of protein interactions derived from risk variants identified from GWAS and statistical summaries conducted among different populations [3, 30, 31].

Moreover, the use of molecular networks has facilitated progress in many areas of biomedical science, such as understanding and linking the functional molecular interactions between proteins with potential targets that could lead to drug development. This straightforward and effective concept allows us to extract the core relationships between host genes and proteins and it also helps to identify and predict interactions between drugs, study the comorbidity of disease, and discover essential associations. Thus, we constructed the PPI networks for risk proteins associated with severe COVID-19 and inferred the hidden interaction based on disease mapping and GO functional analysis.

Our results are in general agreement with previous association studies of host genetic variants related to severe symptoms during COVID-19 infection. We showed that the identified genetic risk variants and their molecular functions are involved in several activities, mainly related to the immune system, along with notable relationships to the metabolic and hematologic systems. These systems react to viruses and prevent the host from developing severe symptoms during infection, thus the risk variants could lead to multi-organ dysfunction since these systems are involved in the activities of numerous genes expressed in various organs in the human body. Following SARS-CoV-2 infection, the host body activates a complex regulatory system of innate responses to defend against the virus. We found that 14 out of the 22 PPI networks influence cytokine and chemokine signaling; for example, risk factors in the pro-inflammatory cytokine genes *IL-6* and *IL-10* contribute to the process of pathological pain activities in the immune system. In addition, some risk proteins such as *APOE*, *CD55*, *C4BPA*, and *CFH* are involved in severe changes in the interferon alpha/beta signaling pathways, which play a vital function in the host immune response to viruses and help to prevent SARS-CoV-2 infection.

Although the majority of biological pathways and activities between the constructed networks are related to the immune system, we observed noticeable biological pathways that negatively contribute to the metabolic and hematopoietic systems, and development of multi-organ dysfunction such as heart attack, liver and kidney injury, and lung inflammation. We found that 6 out of 22 constructed networks are centrally related to the renin-angiotensin-aldosterone system (RAAS) that regulates the production of the hormone angiotensin II. This hormone binds to its receptors in the human host tissue and has various impacts on several organ activities, such as stimulating vasal construction in the arterioles and promoting sodium reabsorption in the kidneys [32].

Moreover, the RAAS contributes to the central nervous system, which suggests that angiotensin II could have effects on the brain. For example, angiotensin II stimulates thirst by acting on the hypothalamus. Furthermore, angiotensin II reduces the baroreceptor response to increased blood pressure, so that this response would not counteract the effect of RAAS. Overall, the effects of the RAAS on metabolic and blood systems lead to increased blood volume and pressure. Also, some of the risk proteins for severe COVID-19 are present in components of the RAAS that are used as clinical biomarkers related to the hematopoietic system. In addition, some biomarkers of RAAS such as *ACE* are affected by risk variants located in *ACE2* [33, 34]. These risk variants downregulate *ACE2* expression, which affects the *ACE* inhibitor and bradykinin pathways and increases activities of multiple pathophysiological pathways that contribute to cardiovascular disease. Furthermore, hypertension is caused by inappropriate activation of the RAAS. Hence, this system is often a target for antihypertensive drugs and the levels of some RAAS proteins are used as medical blood markers during diagnosis of COVID-19 [35–37]. Thus, taking genetic risk factors into account during the diagnosis of patients with COVID-19 could help to prevent organ complications by enabling future complications to be predicted at an early stage based on genetic analysis of risk factors. This suggestion is based on our deep systematic analysis workflow of all genetic risk factors associated with severe COVID-19 from scientific articles and medical reports published during the last two years. Overall, our enrichment analysis and construct PPI networks of genetic risk factors. associated with severe COVID-19 support the value of personalized medicine approaches to predict future complications and organ dysfunction during or after infection among patients with COVID-19.

## Conclusion

In conclusion, our study highlights the potential of studying the molecular functions and interactions of genetic risk factors to identify significant biological mechanisms and pathogenicity pathways that are involved in the development of severe COVID-19 outcomes. Moreover, the study emphasizes the importance of inferring the hidden interactions between networks based on the disease and functional similarity between the networks. We found most of the pathways discovered, and hence the associated risk factors, are mainly related to the immune system with a notable number of pathways related to the metabolic and cardiovascular systems. This evidence reveals the genetic risk factors associated with COVID-19 are involved in several pathways that cause multi-organ dysfunction in different systems. This work underscores the importance of analyzing the molecular interactions and pathways between the SARS-CoV-2 virus and the host to understand the heterogeneous susceptibility of the immune response. This study proposes new insights into pathogenicity analysis of infections by including risk genetic information as essential factors to support individualised clinical treatment plans and predict future complications during and after infection.

## Materials and methods

We designed a computational workflow to apply a deep systematic analysis of identified variants related to severe COVID-19 outcomes that were reported in 35 published works from December 2019 to 2021. These 35 studies were identified from the PubMed search engine using keywords queries such as “Severe-COVID-19”. The 125 variants were retrieved from these genetic studies using text mining algorithms and we validated the final list of risk variants manually. The list of risk variants passed through four phases: deep enrichment analysis of the molecular functions and pathways the risk factors are involved in, and construction of molecular networks to understand the pathogenicity and mechanisms that lead to severe COVID-19 outcomes.

### Characteristic of retrieved dataset of COVID-19 risk factors

From GWAS studies of all loci in the host human genome with a sample size more than or equal 500 [3, 7, 24, 26–28, 30, 38, 39], we retrieved and listed the genetic variants associated with severe COVID-19 in any population with *P* values $< 5 \times 10^{-5}$ or risk variants mentioned as significant in review studies. Most of the retrieved articles were case-control studies of the genome sequences patients with COVID-19. The papers included patients of Chinese, European, Middle-Eastern, and other ethnicities, with different symptoms and comorbidities, with an approximate average age of 63 for males and 58 for females. The youngest patient was only two-years-old, and the oldest was an 85-year-old man. Most papers classified the patients into six levels of severity: asymptomatic, mild, moderate, severe, critical, and deceased. Two articles conducted GWAS with controls who had mild/moderate symptoms to avoid type II error. Most variants identified in GWAS or statistical analysis had 95% significance and above. However, not all retrieved papers mentioned the *P* values for the identified variants. Since the final number of filtered variants was relatively small and not all papers indicated *P* values, we neglected the condition of significance. In our analysis, we called each variant causally associated with severe COVID-19 a risk variant and its host gene, a risk gene. Furthermore, we called the proteins encoded by risk genes risk proteins.

### Computational workflow

**Fig. 12:**
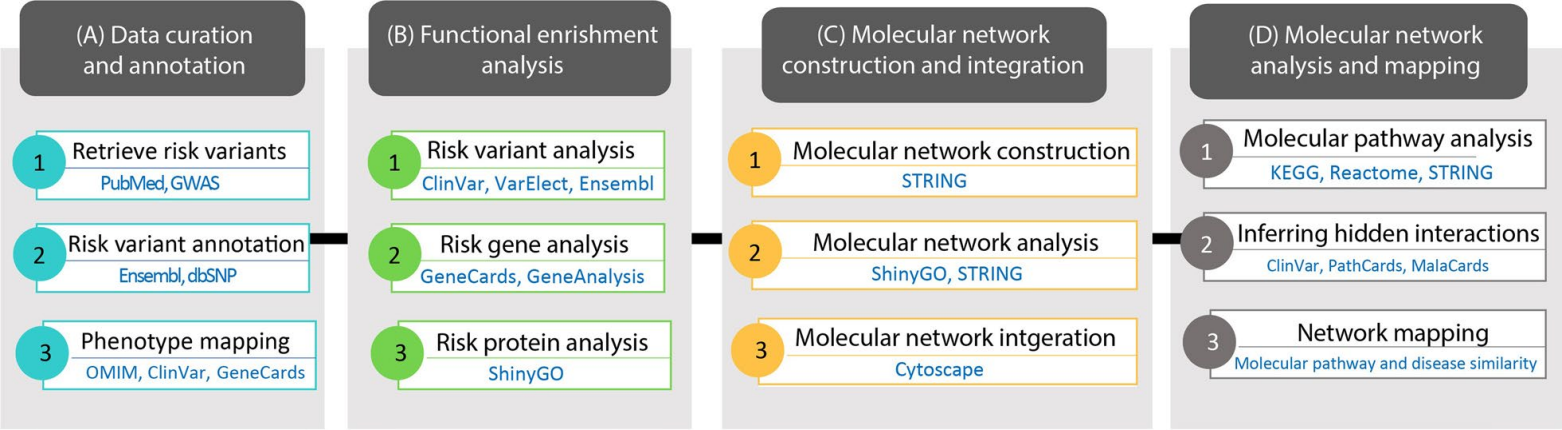
Computational network analysis workflow. The four-stage implemented workflow: (A) data curation and annotation, (B) functional enrichment analysis of risk factors, (C) molecular network construction and integration of risk factors, and (D) molecular network analysis and mapping

A general overview of the phases involved in our computational network analysis workflow is shown in Fig. 12. Our workflow contains four phases: data curation and annotation, functional enrichment analysis of risk factors, molecular network construction and integration of risk factors, and molecular network analysis and mapping based on related diseases and the similarity of ontologies of the risk factor pathways and their molecular functions.

#### Phase 1: data curation and annotation

In phase one, we retrieved 125 risk genetic variants associated with severe COVID-19 from GWAS studies and statistical genetic studies based on GWAS reports. After quality control and removing duplicates, 109 risk variants were annotated using known databases. The dbSNP database [40] was used to identify the chromosomal location of the risk variants based on the CRCh 38 human reference genome for consistency purposes and Ensembl [41] was used to retrieve genomic information and the types of the variants.

In addition, the profiles of the 109 genetic risk variants were mapped to 60 genes using the GeneCards platform [42, 43]. Then, the risk variants were mapped to related diseases using ClinVar [44], Online OMIM [25], and MalaCards databases [45] to complete the profile of each variant to give intensive information.

The curated dataset of genetic risk factors for severe COVID-19 contains 109 risk variants, 60 risk genes, and 56 proteins, and other features that provide a fingerprint of each risk factor related to the severity of COVID-19. Eleven annotations were used to describe the risk variants, including the main features (rs ID, chromosomal location, functional consequence, host gene, *P* value, related diseases, and references). The curated dataset is provided in Additional file 2: Table S1.

#### Phase 2: functional enrichment analysis of risk factors

In phase two, enrichment functional analyses were applied on our curated dataset using Gene Ontology (GO) [46] on the risk variants, genes, and proteins to obtain enriched information describing the molecular functions and processes of the genetic risk genetic factors related to severe COVID-19.

In terms of risk variant analyses, we used the Ensembl [41] and VarElect [47] platforms to identify the structure and functional consequences of the risk variants to obtain the functional distribution and characteristics of the risk variants.

At the risk gene level, we applied expression-based analysis using GeneAnalytics [48], which relies on LifeMap Discovery [49] to identify gene associations with tissues and cells. The matching scores for gene expression in different normal tissues and cells were calculated with a search for maximum similarity between expression vectors [48]. The ranked scores for enriched risk genes were calculated using LifeMap discovery by integrating gene annotations from scientific publications, as well as bioinformatic data. A matching score was assigned to each risk gene based on their annotations in the data. The overall score depends on the number of matches for each risk gene. Moreover, we assessed the matching quality by categorizing the results as high-, medium-, and low-quality matches; only high-quality matches were considered in the analysis. The distribution of the matching qualities across the list of results is also presented to provide an easy assessment. Then, we applied functional enrichment analysis to the list of risk genes related to severe COVID-19 outcomes using GO terms and GeneAnalytics [40, 48]. We used ShinyGo [50] to analyze the ontologies of the compartments and tissues of the risk genes and associated diseases based on a 0.05 FDR cutoff and minimum and maximum pathway size of 2-2000.

In terms of risk protein analyses, we used STRING 11.5 [51] and ShinyGo [50] to identify molecular functions and biological pathways associated with the 56 risk proteins based on a threshold of FER equal 0.05.

#### Phase 3: molecular network construction and integration

In phase three, we constructed and analyzed the PPI networks of the 56 risk proteins using the STRING 11.5 database [51, 52]. All constructed networks are full STRING networks, which means the edges between proteins indicate both functional and physical associations. Having a functional association means that the proteins have the same function, while having physical associations indicate common molecular components that link the proteins. We considered all active sources of protein interactions that are reported in the literature, validated experimentally, and retrieved from public databases such as GeneCards for gene interpretations, Reactome [53], and Kyoto Encyclopedia of Genes and Genomes (KEGG) [54, 55] for gene pathways.

Furthermore, all retrieved interactions satisfy the criterion of having an interaction score of at least 0.9 to guarantee that the associated interaction is sufficiently significant to enhance further analysis. We constructed the top ten interactions for each risk protein based on the highest significant *P* values among all PPI candidates to limit the network outputs and avoid unclean networks with unnecessary interfering links for more effective network visualization.

Then we integrated the PPI networks constructed for the 56 risk proteins with the 109 risk variants. Visualization of the integrated data using Cytoscape [56] generated 24 PPI networks and seven orphan proteins. Then we mapped the unconnected networks based on their common risk variants or the similarity of their molecular functions using GO, STRING database [51, 52], and GeneCards.

#### Phase 4: molecular network pathway analysis and disease mapping

Finally in phase four, we analyzed the molecular network pathways to identify pathogenicity and pathways that related to severe outcomes of COVID-19 using Reactome [53] and the Kyoto Encyclopedia of Genes and Genomes (KEGG) [54, 55]. In addition, we mapped the correlation between disease-variant-network to find similarity and shared biological pathways between related disease and the molecular functions of the constructed networks using the ClinVar [44], PathCards [57], and MalaCards platforms. Then, we linked the outputs to infer the hidden connections between the constructed networks based on disease mapping, network molecular functions, gene ontology analysis, and pathway similarity.

## Supplementary Information

**Additional file 1.** Details of estimating genetic effects of severe COVID-19 outcomes.

**Additional file 2.** List of the 109 genetic risk variants and 24 network mapping for severe COVID-19.

**Additional file 3.** Enrichment analysis of the molecular functions of the 24 constructed networks.

**Additional file 4.** Details of the molecular pathways of the 24 constructed protein–protein interaction networks.

## Data Availability

The curated dataset of genetic risk factors of severe COVID-19 is provided in the Additional file 1.

## Abbreviations

SARS-CoV-2: Severe Acute Respiratory Syndrome Coronavirus 2
COVID-19: Coronavirus 2019 disease
WHO: World Health Organization
CDC: Centers of Disease and Control Preventions
EWAS: Exome-Wide Association Study
GWAS: Genome-Wide Association Study
MR: Mendelian randomization
COLOC: Bayesian colocalization
PheWAS: Phenome-Wide Association Study
eQTL: Expression quantitative trait loci
SNPs: Single nucleotide polymorphisms
TWAS: Transcriptome-Wide Association Study
WGS: Whole genome sequencing
eQTLGen: Gene expression quantitative trait loci
pQTL: Protein quantitative trait loci
3-UTR: $3^\prime$ Untranslated region
FDR: False discovery rate
PPI: Protein–protein interaction
TF: Transcription factor
GO: Gene ontology
KEGG: Kyoto encyclopedia of genes and genomes
MHC: Major histocompatibility complex
bHLH: Basic helix-loop-helix
SLE: Systemic lupus erythematosus
AGS: Aicardi–Goutières syndrome
BLS1: Bare lymphocyte syndrome, type I
ASCVD: Development of atherosclerotic cardiovascular disease
CAD: Coronary artery disease
VTE: Venous thromboembolism
TSH: Thyroid-stimulating hormone
MCHC: Mean corpuscular hemoglobin concentration
RBC: Red blood cell
HUS: Hemolytic uremic syndrome
LQTS: Long QT syndrome
ICH: Intracerebral hemorrhage
PTH: Parathyroid hormone
RTD: Renal tubular dysgenesis
RCA: Regulator of complement activation
RAAS: Renin–angiotensin–aldosterone system
