# Supplementary material for: Computational network analysis of host genetic risk variants of severe COVID-19
Source: Hum Genomics. 2023 Mar 2;17:17. doi: 10.1186/s40246-023-00454-y (PMC9977643; doi:10.1186/s40246-023-00454-y)
Supplement: Supplementary file 1 — Additional file 1. Details of estimating genetic effects of severe COVID-19 outcomes. [file 40246_2023_454_MOESM1_ESM.pdf]

# Statistical analysis of curated dataset of severe COVID-19 risk factors

The association between the MAFs and genetic effects of risk variants of severe COVID-19 is shown in the following plot.

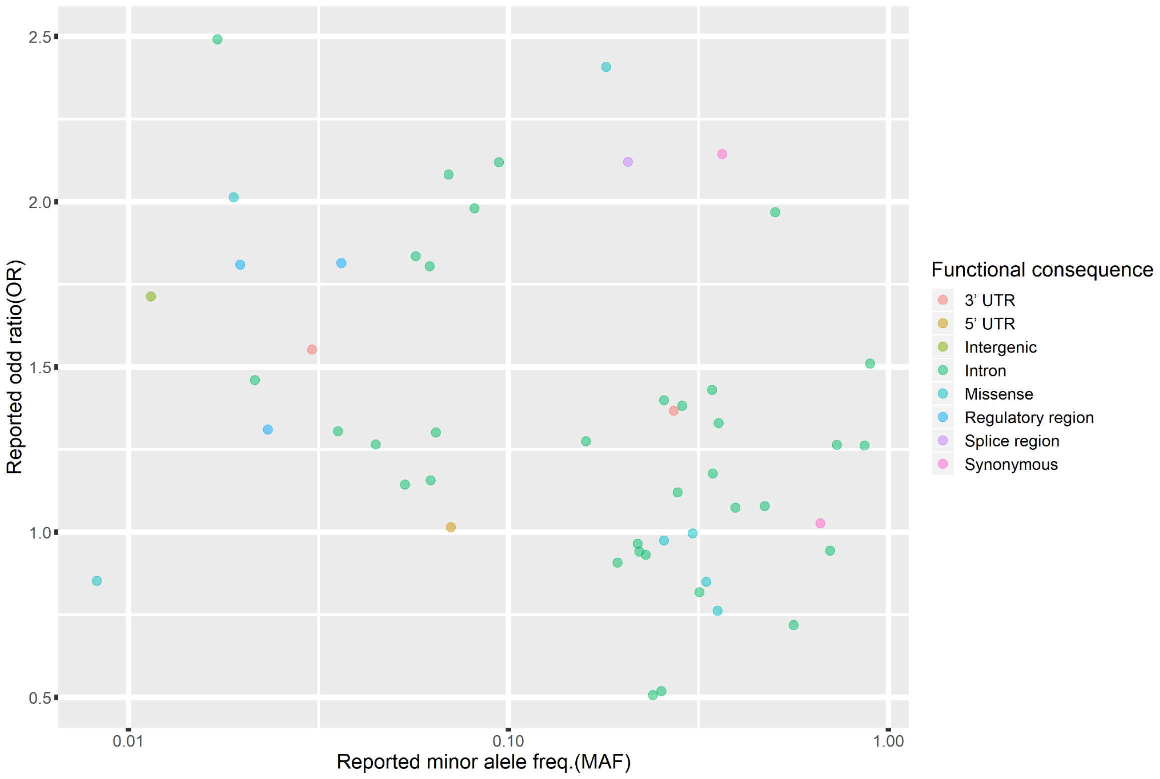


Fig. 1 The genetic effects of risk variants associated with severe COVID-19 outcomes

# Estimation of the additive effect of risk variants per risk gene

The association between each risk variant and its effect in developing severe outcomes of COVID-19 was established using an additive genetic model estimating the OR of severe COVID-19. We fit a linear model to estimate the genetic effect based on new weighted OR (WOR) and MAF per gene. The coefficient of risk gene effect was added to the linear model to estimate the most influential risk genes without considering the cumulative number of risk variants per gene. Fig.2 The forest plot illustrates the top 30 risk genes with significant P-values < 0.005. Although the genes are significant in terms of P-value, their effect on severe COVID-19 is quite low.

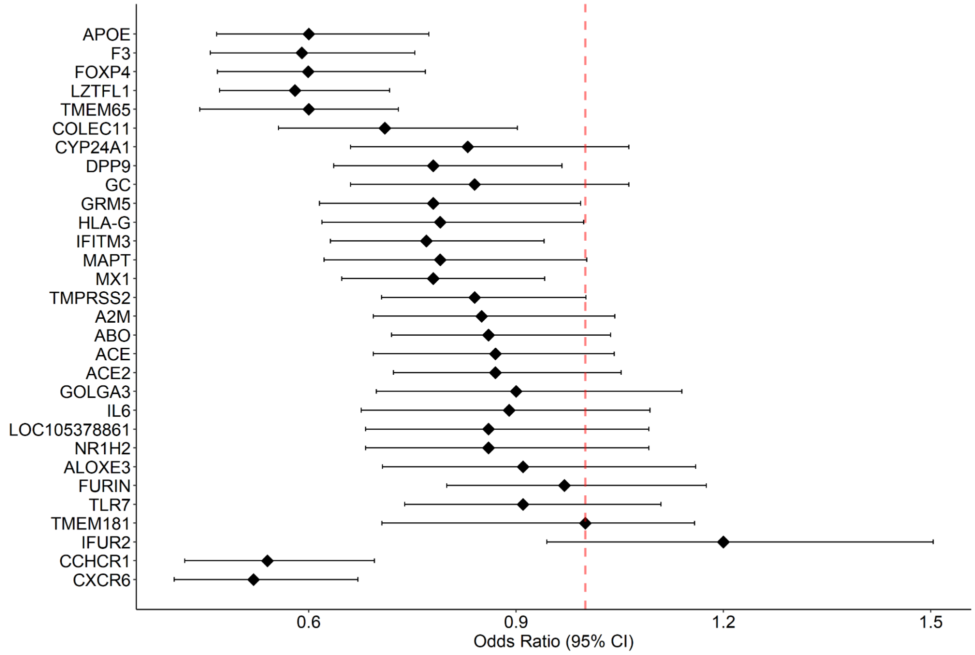


Fig. 2 The additive effects of risk genes on severe COVID-19 outcome. The top 30 risk genes associated with severe COVID-19 outcomes with significant P-values < 0.005.

.

Thus, we consider that some of the risk genes contain more than one risk variant with different effects. In order to calculate their additive effects, the cumulative value of reported OR and MAF of the number of accumulated risk alleles in a particular gene was calculated and used as a combined effect to estimate the additive effects per gene. The scatter plot in Fig.3 shows the additive effects of risk variants on severe COVID-19 outcomes per gene. We calculated the additive effects of the identified risk variants associated with severe COVID-19 outcomes for all genes. The following scatter plot shows the additive effects of risk variants on severe COVID-19 outcome per genes. Each point in the scatter plot corresponds to the additive effect of the risk gene that has been calculated based on cumulative values of reported ORs and MAF. Each gene hosts at least one reported risk variant.

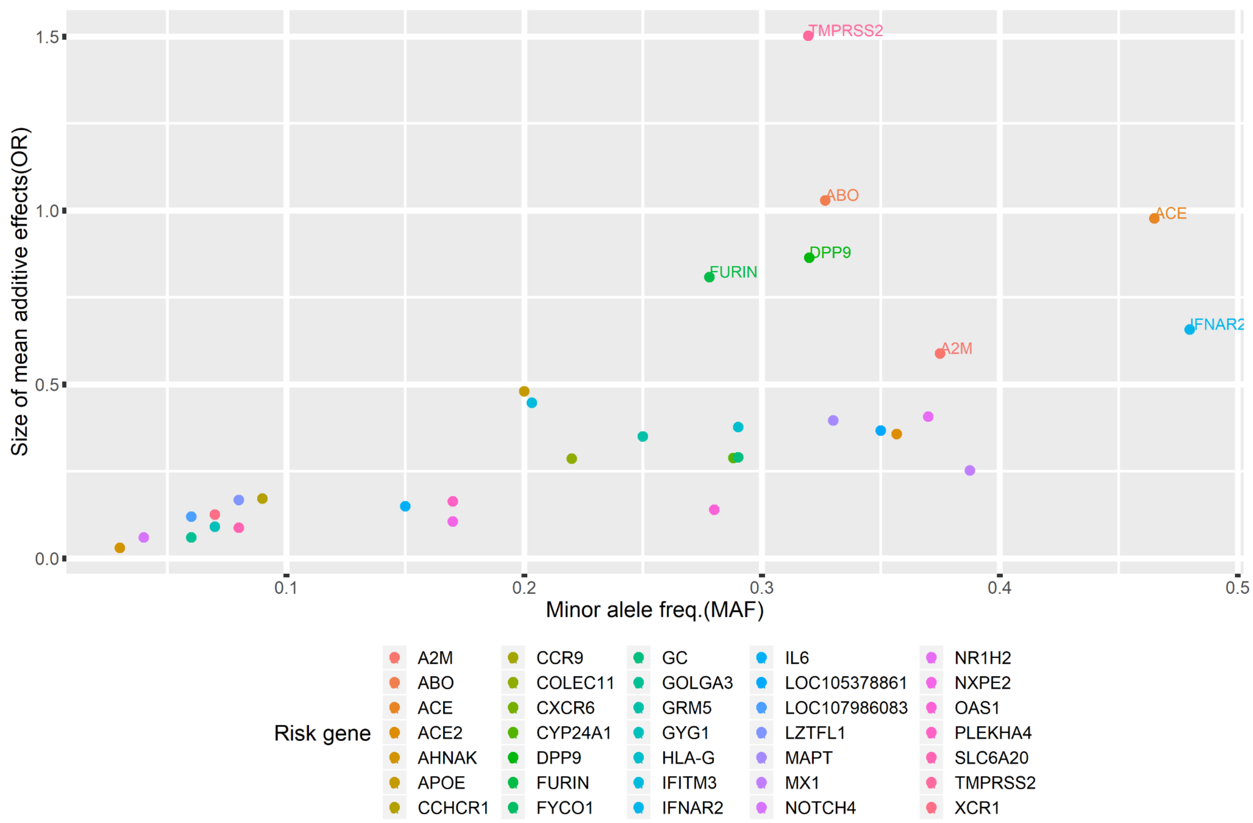


Fig.3 The additive effects of risk variants on severe COVID-19 outcome per genes. The scatter plot shows the additive effects of risk variants on severe COVID-19 outcomes per gene. Each point corresponds to the additive effect of the risk gene that has been calculated based on cumulative values of reported ORs and MAF. Each gene hosts at least one reported risk variant.

## Statistical methods

### Weighted sum statistics method of odd ratio

However, we calculate the OR Weighted Sum Statistics (ORWSS) for the case-control design study which does not require a threshold for defining rare variants, we consider a similar sequence of different weights of OR as reported in (Hoffmann, Marini and Witte, 2010), (Feng, Elston and Zhu, 2011). We applied the weighted threshold method:

$$w_i = \{\gamma_i\,,\ if\ \gamma_i > \bar{\mu} + c\sigma\ or\ \gamma_i < \bar{\mu} - c\sigma\,,\ 0\ otherwise\}$$

Where $w_i$ is the generated weight of OR, $\gamma_j$ is the reported odd ratios that represent the effects of risk variants on COVI-19 severity based on the literature. The $\bar{\mu}$ is the mean from the variant effects $\gamma_i$, $i = 1, \ldots n$, and t σ.is the standard deviation of the variant effects. The constant $c$ is a pre-specified parameter to scale different weight in terms of OR.

For example, the calculated weighted odd ratio (WOR) shows that *A2M* and *ACE* have more effects than other genes.

Table 2: An example of weighted odd ratio (WOR) from curated dataset

| Risk gene | # of risk variant | Cum. OR | Cum. OR | Cum. MAF | WOR |
|---|---|---|---|---|---|
| A2M | 2 | 1.57 | 1.57 | 0.75 | 1.16 |
| ABO | 6 | 3.15 | 3.15 | 1.96 | 0.85 |
| ACE | 2 | 2.1 | 2.1 | 0.93 | 1.51 |
| ACE2 | 3 | 0 | 0 | 1.07 | 0.36 |
| AHNAK | 1 | 0 | 0 | 0.55 | 0.55 |
| MX1 | 4 | 1.8 | 1.8 | 0.56 | 0.59 |

The list of weighted-OR and genetic effects of each risk variant on the severity of COVID-19 is added as Column D-I to Table 6 in Additional File 1. The following paragraph has been added to the manuscript in the result section at line 149:

"Several methods estimate different weights of OR and calculate genetic risk scores. We calculated the OR Weighted Sum Statistics (ORWSS) for a case-control design study that does not require a threshold for defining rare variants [18,19]. The list of weighted-OR and genetic effects of each risk variant on the severity of COVID-19 is displayed in Table 6 in Additional File 1. Moreover, the statistical method of estimating the genetic effects of severe COVID-19 is explained in Additional File 4"

In terms of genes, we fit a linear model to identify the significant genes that have more effects on severe COVID-19 outcomes based on the reported and generated effects. applied the following alternative additive genetic model:

$$g\left(Y_i\right) = \alpha_0 + \gamma + \sum_k w_k X_{ik}$$

Where $Y_i$ is the effect of risk genes associated with COVID-19 outcomes, $k$ is the number of variants contained in $Y_i$. and $\gamma$ is a vector of combined genetic effects of aggregate multiple rare variants and leverages their combined strength to improve estimation of the effect of a weighted combination of variants. The $w_k$ is the original effect weight of identified variant, the reported odd ratios that represent the effects of risk variants on COVI-19 severity based on the literature, $X_{ik}$ is the MAF of the variants.

## Estimating additive genetic effects

Since our dataset is constructed by retrieving available genetics information related to the identified risk variants associated with COVID-19 severity from the literature, the dataset contains some missing entries since not all

reviewed papers provide the same amount and types of information. For example, most of the reviewed papers do not provide the genotype distribution of the 2 × 2 genotype matrix for their findings. Thus, we relied on the odd ratio (OR) and the minor allele frequency (MAF) of each risk variant to calculate a cumulative effect that represents the additive effects of variants per gene as an alternate method. We applied the following alternative method to calculate the additive effects a:

$$Y_i = \alpha_0 + (\sum_k w_k x_{ik})$$

Where $Y_i$ is the risk genes associated with COVID-19 outcomes,, $k$ is the number of variants contained in $Y_i$. $w_k$ is the original effect weight of identified variant, $x_{ik}$ is the MAF of the variants.

Table 1 is an example of the input data to our method to calculate the additive effects of variants per gene. The following table contains two identified risk genes *LZTFL1* and *A2M* as examples, where the *LZTFL1* gene has three risk variants with different ORs, while *A2M* has two.

Table 1: An example of calculated AE dataset

| Risk gene $Y_i$ | # of risk variants | Risk variant | Reported OR | Reported MAF | Cum. MAF | Cum. OR | AE |
|---|---|---|---|---|---|---|---|
| LZTFL1 | 3 | rs35652899 | 1.2 | 0.05 | 0.9 | 4.5 | 4.1 |
| | | rs76374459 | 1.2 | 0.05 | | | |
| | | rs73064425 | 2.1 | 0.08 | | | |
| A2M | 2 | rs669 | 0.8 | 0.378 | 0.7 | 1.6 | 1.1 |
| | | rs4883215 | 0.8 | 0.365 | | | |

# References

Feng, T., Elston, R.C. and Zhu, X. (2011) 'Detecting rare and common variants for complex traits: sibpair and odds ratio weighted sum statistics (SPWSS, ORWSS)', *Genetic epidemiology*, 35(5), pp. 398–409.

Hoffmann, T.J., Marini, N.J. and Witte, J.S. (2010) 'Comprehensive approach to analyzing rare genetic variants', *PloS one*, 5(11), p. e13584.
