# Supplementary material for: Computational network analysis of host genetic risk variants of severe COVID-19
Source: Hum Genomics. 2023 Mar 2;17:17. doi: 10.1186/s40246-023-00454-y (PMC9977643; doi:10.1186/s40246-023-00454-y)
Supplement: Supplementary file 4 — Additional file 4. Details of the molecular pathways of the 24 constructed protein–protein interaction networks. [file 40246_2023_454_MOESM4_ESM.pdf]

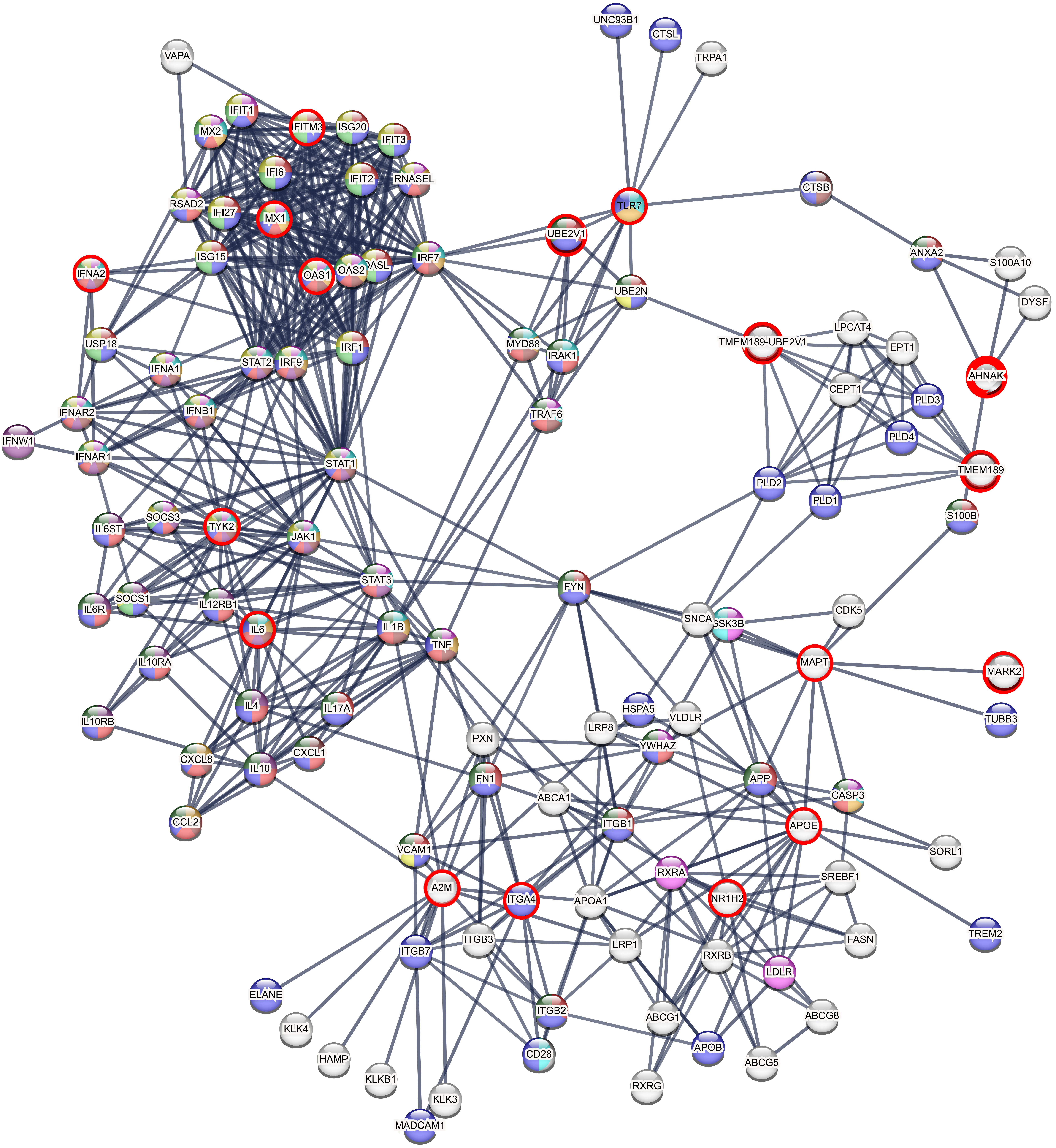

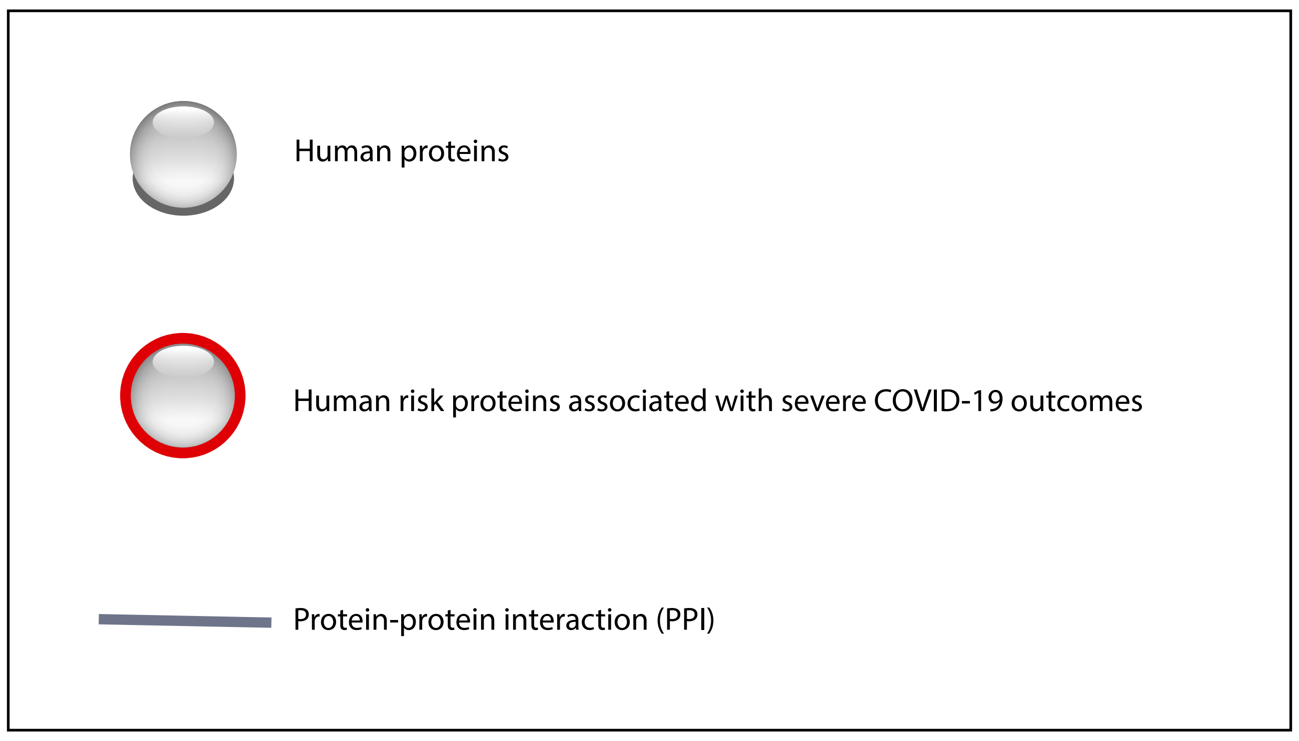

Protein-protein interaction network symbols

Pathways	Description	False discovery rate
HSA-1280215	Cytokine signaling in immune system	1.16e-50
HSA-168256	Immune system	1.87e-44
HSA-909733	Interferon alpha/beta signaling	3.20e-41
HSA-913531	Interferon signaling	1.47e-32
hsa05160	Hepatitis C	7.81e-28
HSA-449147	Signaling by interleukins	2.28e-27
hsa05162	Measles	2.42e-26
hsa05164	Influenza A	3.04e-26
hsa04621	NOD-like receptor signaling pathways	1.44e-21
hsa04630	JAK-STAT signaling	5.26e-21

Functional enrichments in Network 1

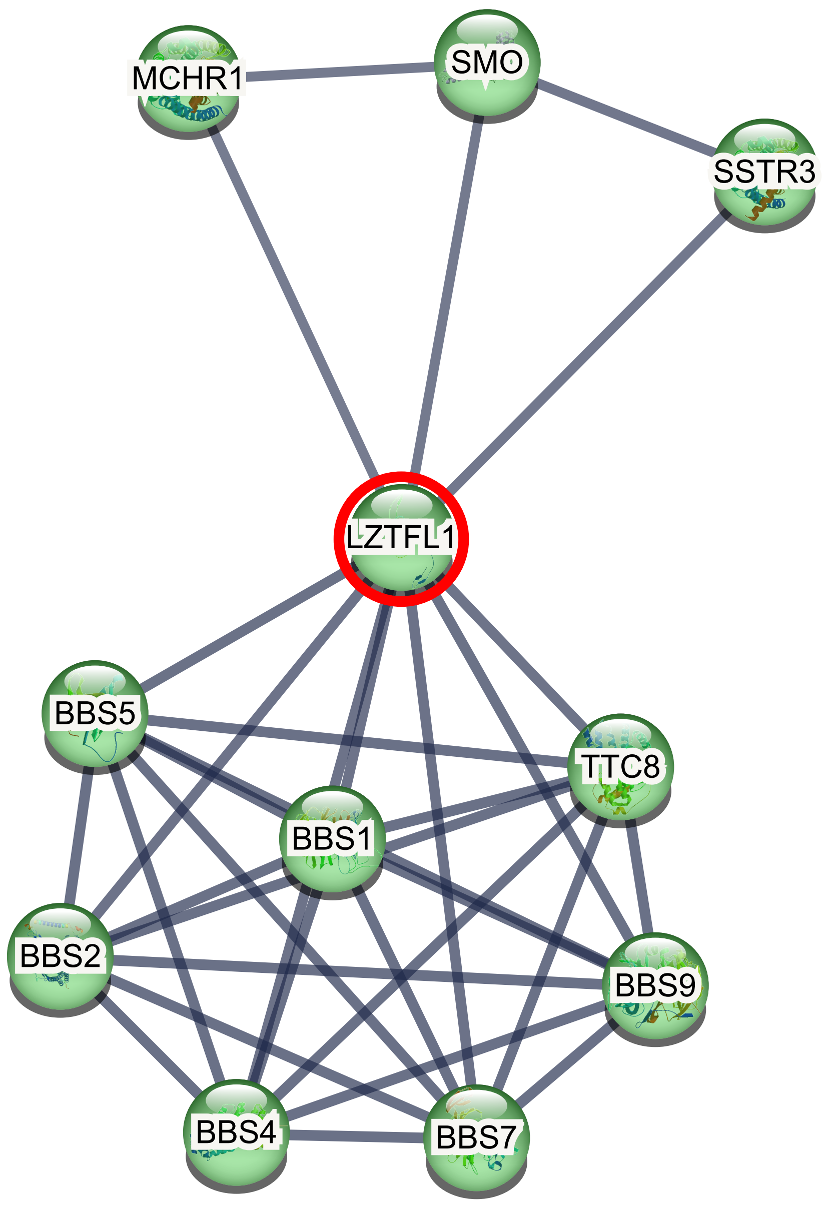

Pathways	Description	False discovery rate
HSA-5620922	BBSome-mediated cargo-targeting to cilium	1.04e-28

Functional enrichments in Network 7

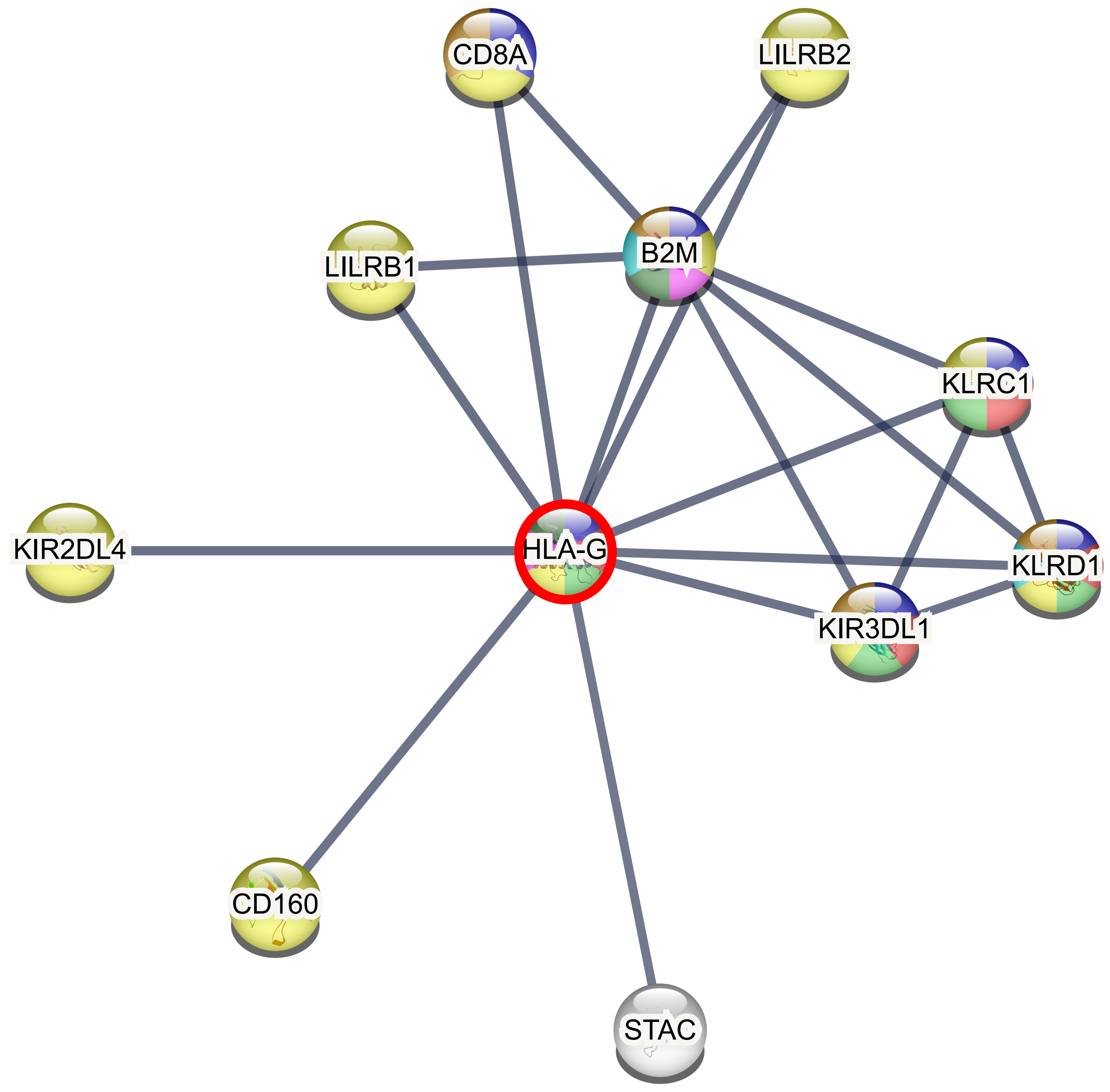

Pathways	Description	False discovery rate
HSA-198933	Immynoregulatory interactions	5.56e-18
hsa04612	Antigen processing and presentation	2.35e-10
hsa05332	Graft-versus-host disease	8.20e-07
hsa04650	Natural killer cell mediated cytotoxicity	5.65e-05
HSA-1236977	Endosmal/vacuolar pathway	0.0121
HSA-983170	Antigen presentation	0.0434
HSA-2424491	DAP12 signaling	0.0448

Functional enrichments in Network 8

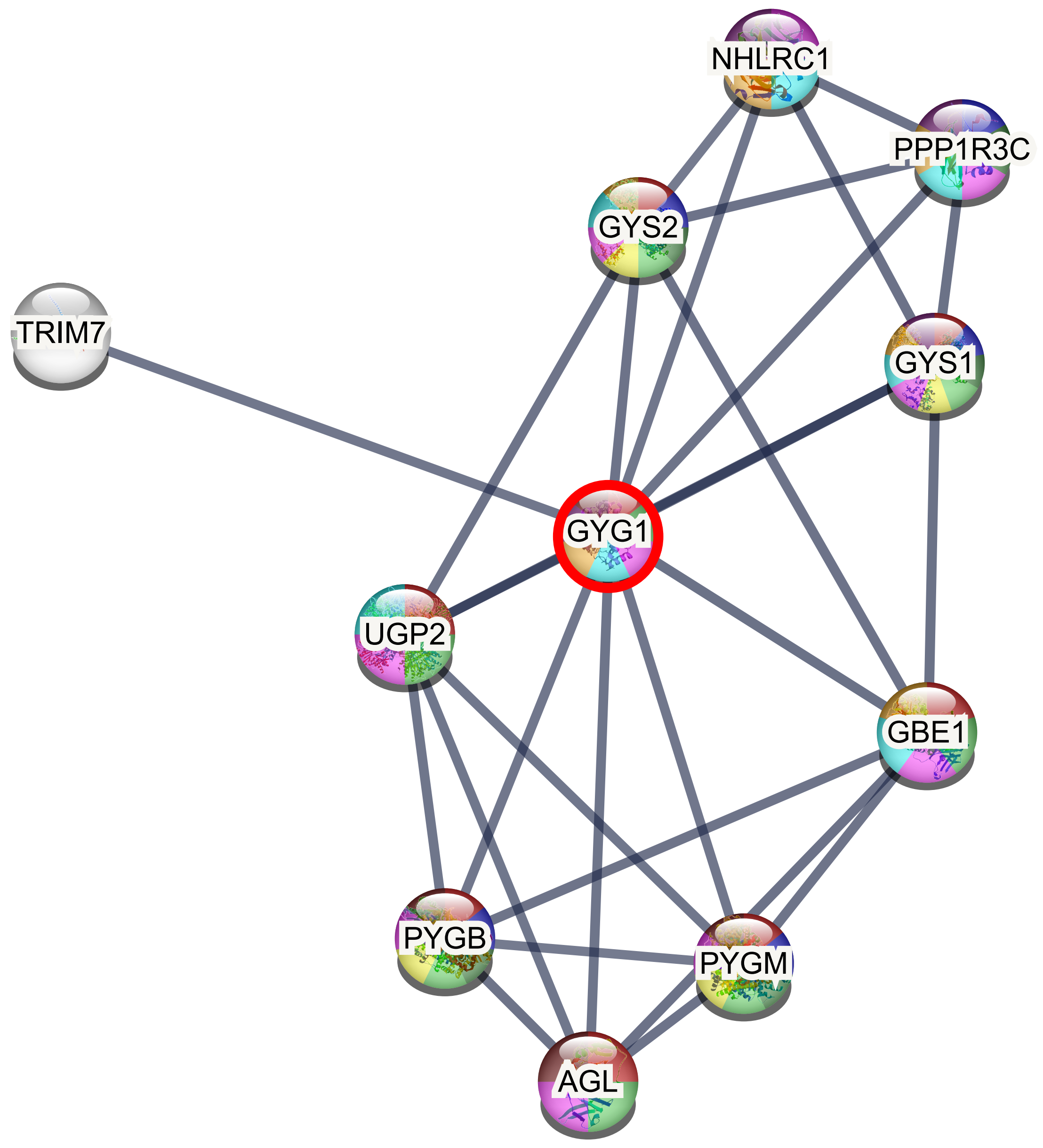

Pathways	Description	False discovery rate
HSA-8982491	Glycogen metabolism	3.66e-24
hsa00500	Starch and sucrose metabolism	7.94e-18
HSA-3322077	Glycogen synthesis	4.01e-16
HSA-3229121	Glycogen storage diseases	2.39e-13
HSA-3785653	Myoclonic epilepsy of Lafora	1.39e-08
HSA-70221	Glycogen breakdown	6.46e-08
hsa04931	Insulin resistance	4.23e-07
hsa04910	Insulin signaling pathway	8.09e-07
hsa01100	Metabolic pathway	1.03e-05
hsa04922	Glucagon signaling pathway	1.68e-05

Functional enrichments in Network 9

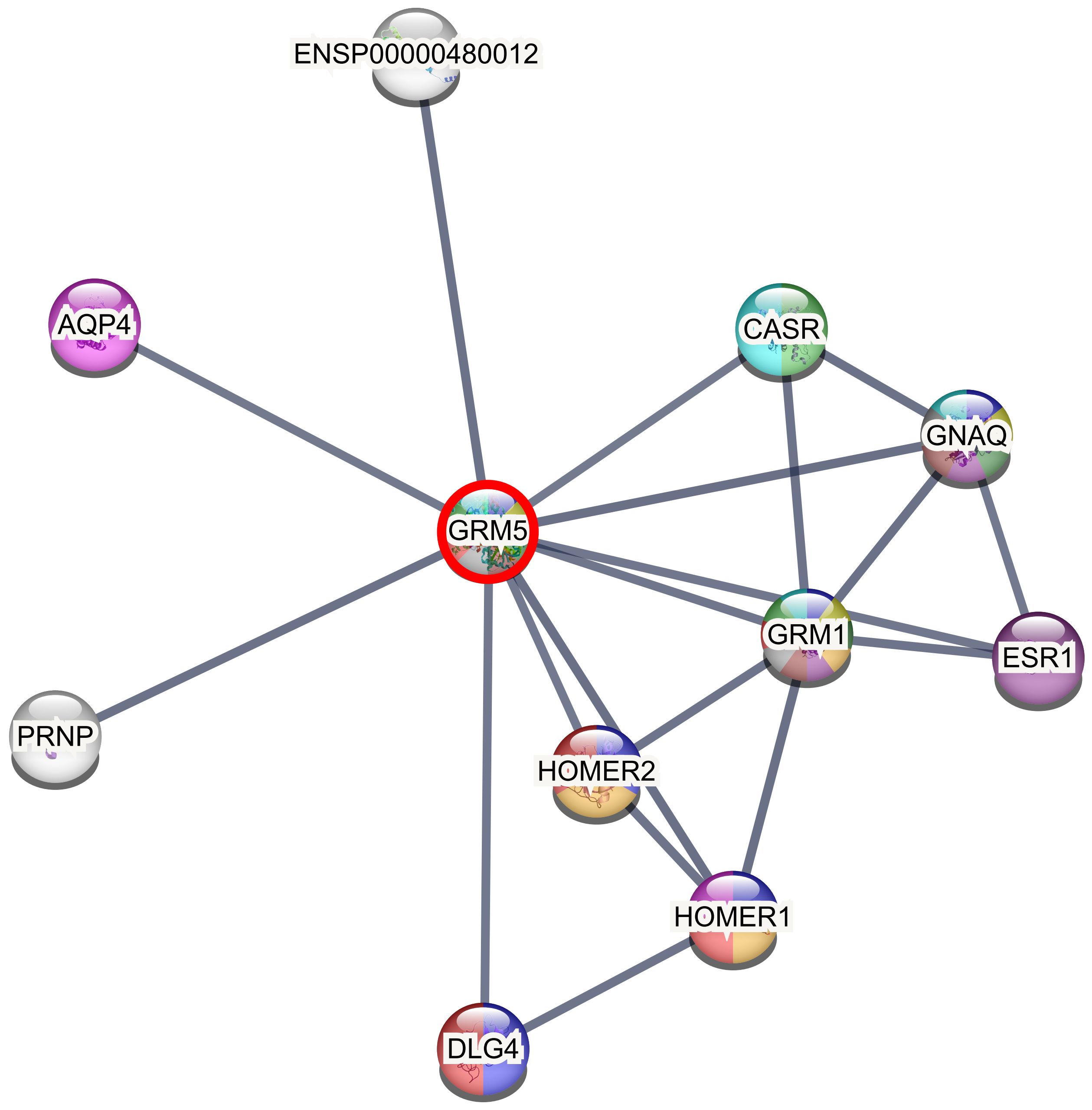

Pathways	Description	False discovery rate
hsa04724	Glutamatergic synapse	6.06e-09
HSA-6794361	Neurexins and neuroligins	2.26e-07
hsa04720	Long-term potentiation	0.0010
HSA-420499	Class C/3 (Metabotropic glumate/pheromone receptors)	0.0011
hsa04540	Gap junction	0.0017
HSA-416476	G alpha (q) signaling events	0.0020
hsa04068	FoxO signaling pathway	0.0038
hsa04915	Estrogen signaling pathway	0.0038
hsa04723	Retrograde endocannabinoid signaling	0.0038
hsa04020	Calcium signaling pathway	0.0074

Functional enrichments in Network 10

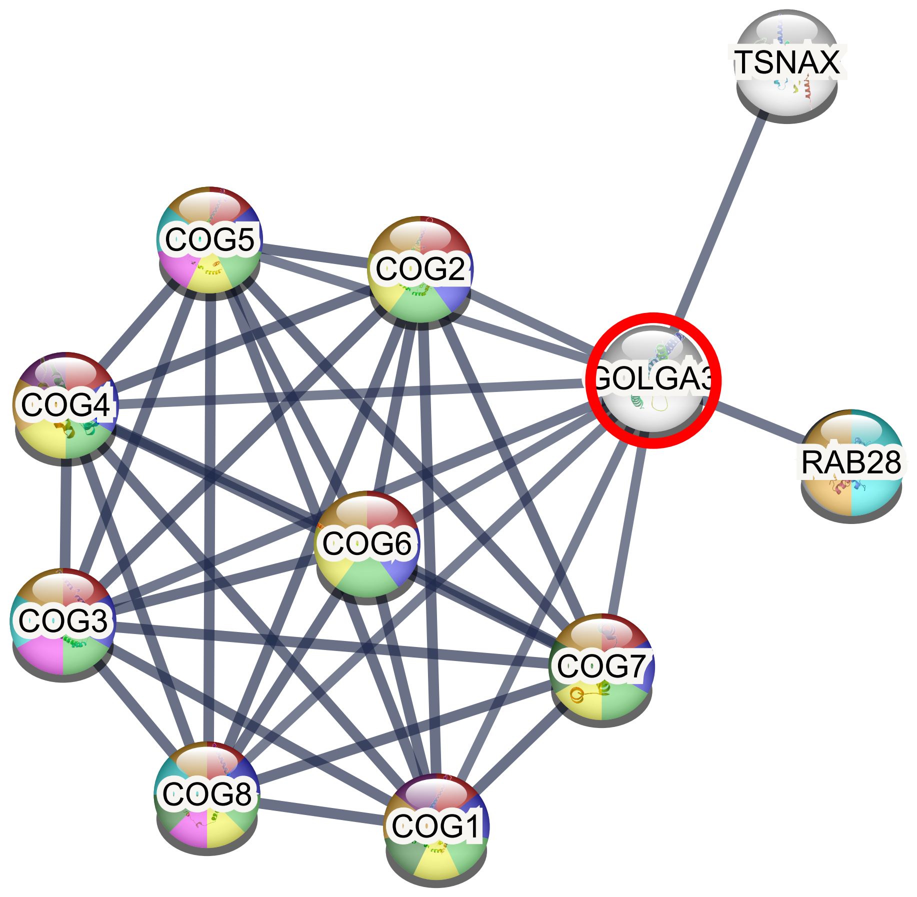

Pathways	Description	False discovery rate
HSA-6811438	Intra-Golgi traffic	4.25e-16
HSA-6811440	Retrograde transport at the Trans-Golgi-Network	5.50e-16
HSA-6807878	COPI-mediated anterograde transpot	7.76e-14
DOID:0050571	Congenital disorder of glycosylation type ii	1.34e-14
DOID:0111006	X-linked cone-rod dystrophy 2	2.88e-06
DOID:0070257	Congenital disorder of glycosylation type iie	4.03e-06
DOID:0050572	Cone-rod dystrophy	1.76e-05
DOID:0050177	Monogenic disease	0.00048
DOID:0070262	Congenital disorder of glycosylation type iij	0.00075

Functional enrichments in Network 11

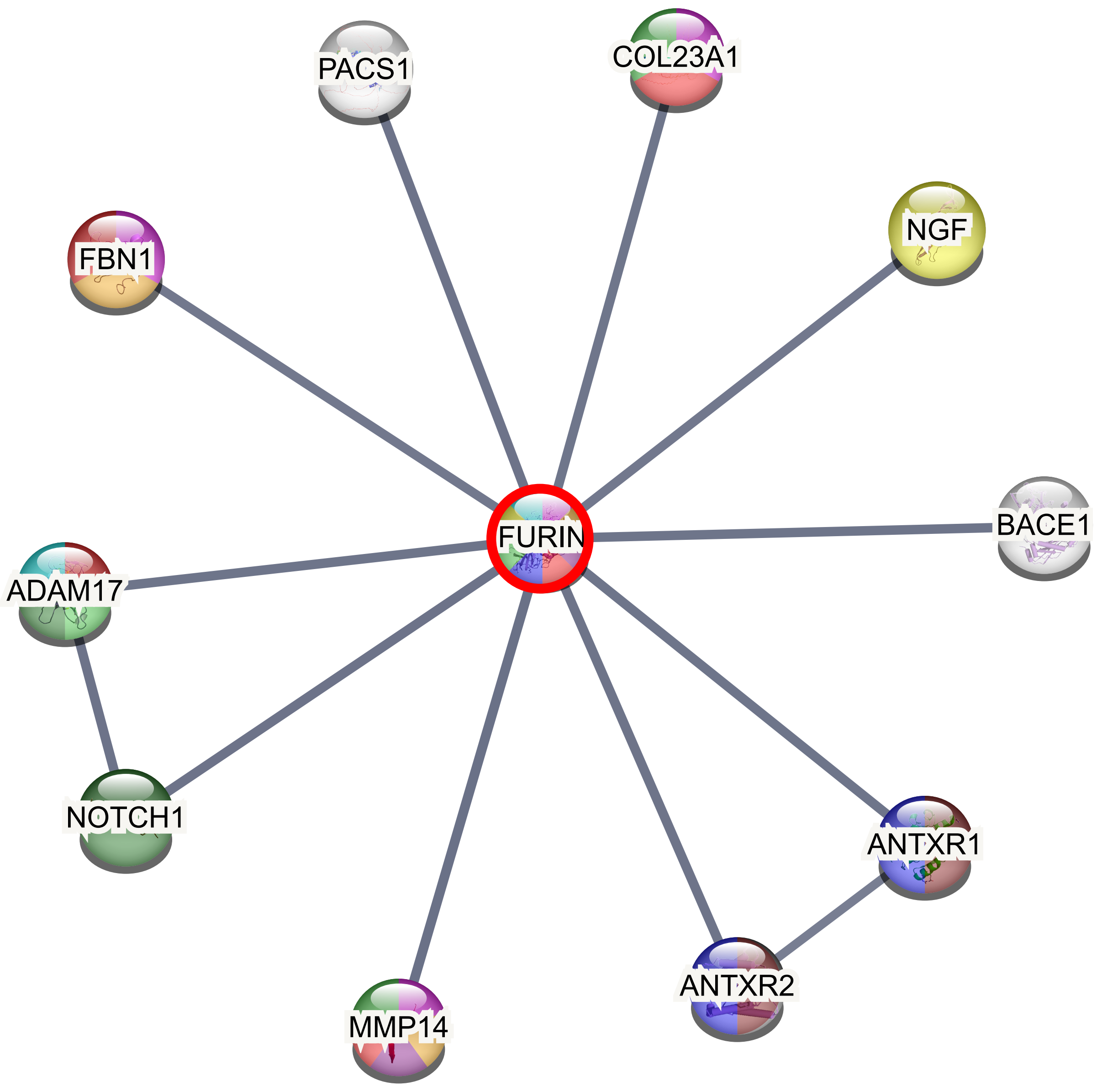

Pathways	Description	False discovery rate
HSA-1474228	Degradation of the extracellular matrix	2.01e-05
HSA-5210891	Uptake and function of anthrax toxins	4.68e-05
HSA-1442490	Collagen degradation	4.683-05
HSA-167060	NGF processing	0.0016
HSA-2660826	Constitutive signaling by NOTCH1	0.0028
HSA-9662834	CD163 mediationg and anti-inflammatory response	0.0034
HSA-2691232	Constitutive signaling by NOTCH1 HD domain mutants	0.0077
HSA-1912420	Pre-NOTCH processing in Golgi	0.0082
HSA-5663205	Infectious disease	0.0082
HSA-1643685	Disease	0.0117

Functional enrichments in Network 12

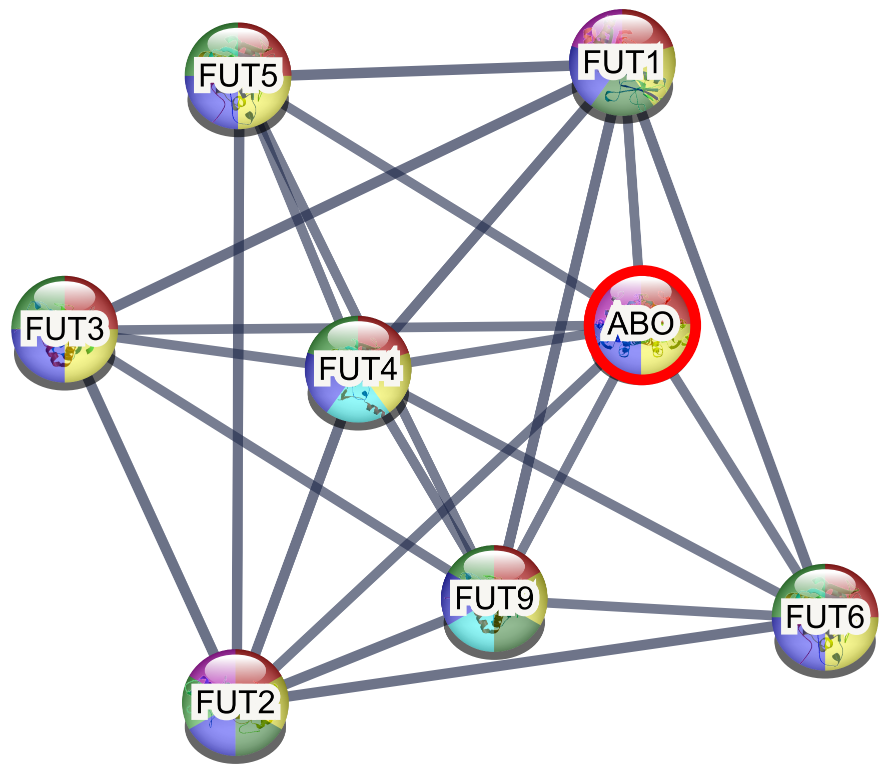

Pathways	Description	False discovery rate
hsa00601	Glycosphingolipid biosynthesis-lacto and neolacto series	1.14e-20
HSA-9033658	Blood group systems biosynthesis	1.26e-20
HSA-9037629	Lewis blood group biosynthesis	3.93e-14
HSA-9033807	ABO blood group biosynthesis	4.86e-07
hsa01100	Metabolic pathways	1.54e-07
hsa00603	Glycosphingolipid biosynthesis- globo and isoglobo series	4.08e-06
hsa00515	Mannose type O-glycanbiosynthesis	0.0034

Functional enrichments in Network 13

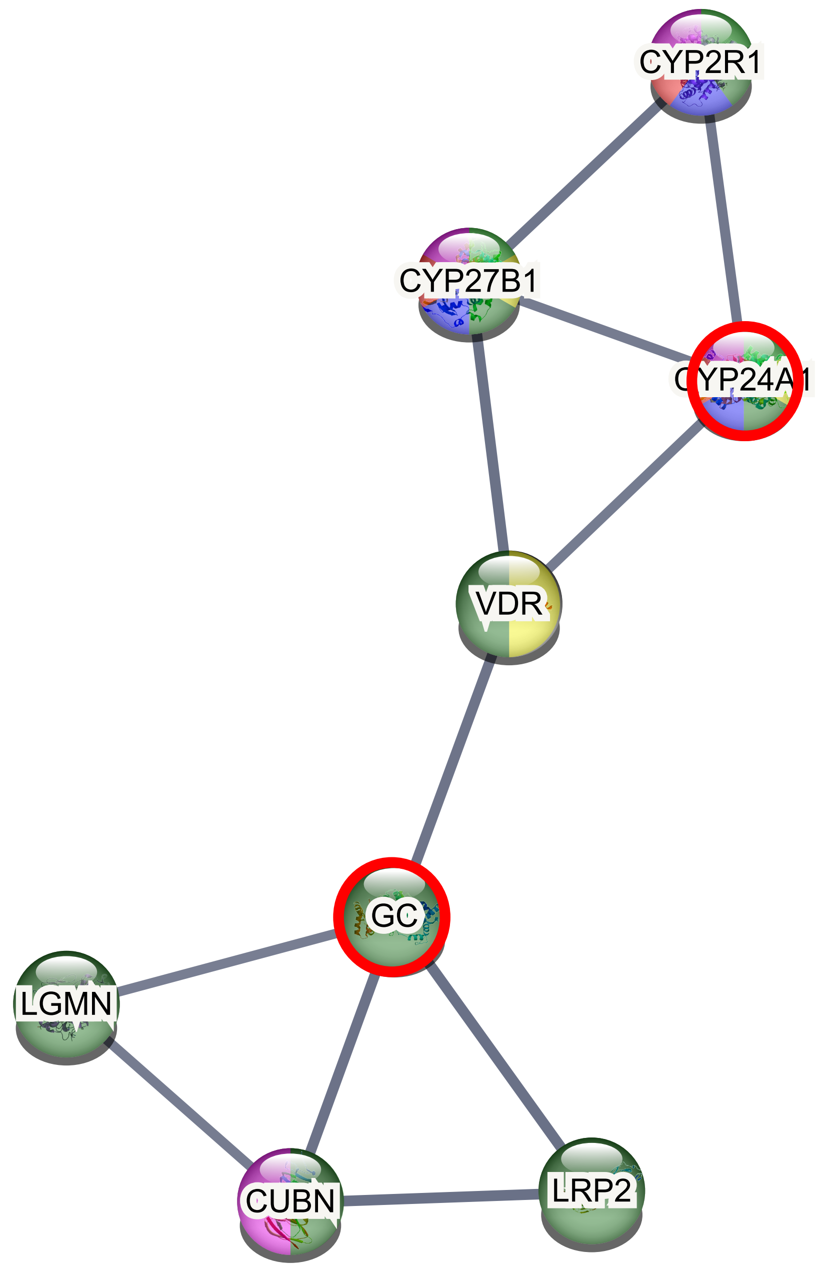

Pathways	Description	False discovery rate
HSA-196791	Vitamin D (calciferol) metabolism	3.08e-22
HSA-211916	Vitamins	3.08e-06
hsa00100	Steroid biosynthesis	2.66e-05
HSA-5579029	Metabolic disorders of biological oxidation enzymes	0.00015
HSA-5668914	Diseases of metabolism	0.00052
hsa04928	Parathyroid hormone synthesis, secretion and action	0.0014

Functional enrichments in Network 14

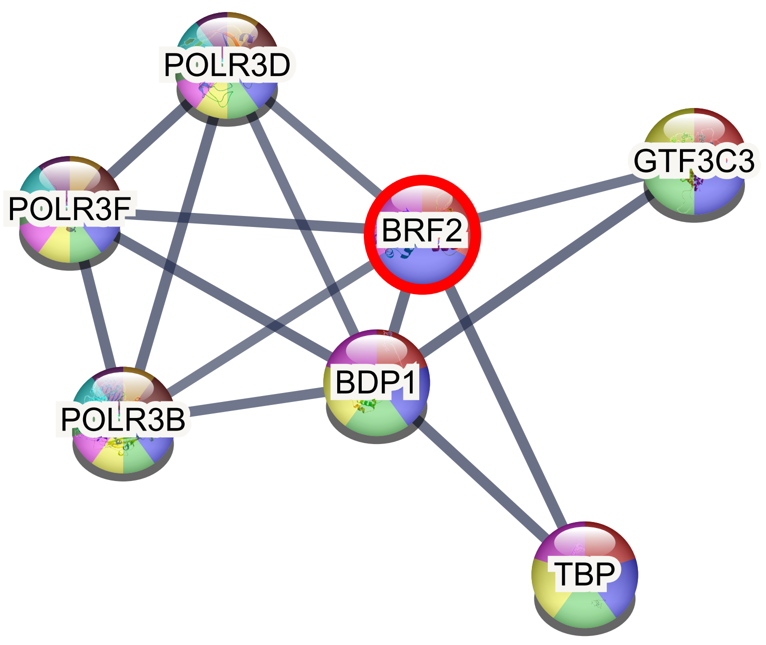

Pathways	Description	False discovery rate
HSA-76046	RNA polymerase III transcription initiation	296e-16
HSA-749476	RNA polymerase III abortive and retractive initiation	3.13e-16
HSA-76066	RNA Polymerase III Transcription Initiation From Type 2 Promoter	4.41e-14
HSA-76061	RNA Polymerase III Transcription Initiation From Type 1 Promoter	4.41e-14
HSA-76071	RNA Polymerase III Transcription Initiation From Type 3 Promoter	4.41e-14
HSA-73780	RNA Polymerase III chain elongation	8.65e-06
HSA-73980	RNA Polymerase III transcription termination	1.55e-05
hsa03020	RNA polymerase	5.61e-05
hsa04623	Cytosolic DNA-sensing pathway	0.00020
HSA-183949	Cytosolic sensors of pathogen-associated DNA	0.00026

Functional enrichments in Network 15

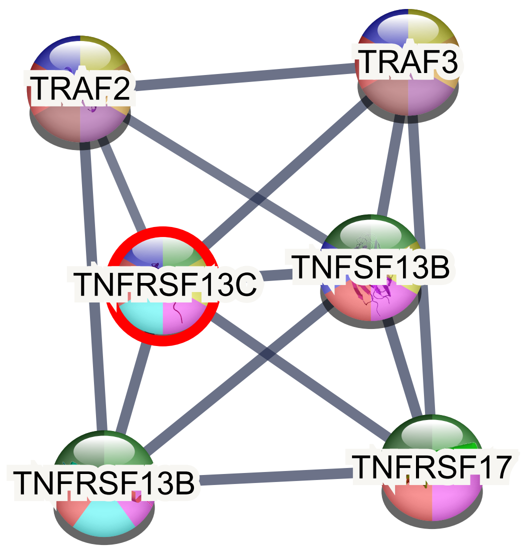

Pathways	Description	False discovery rate
HSA-5668541	TNFR2 non-canonical NF-kB pathway	4.22e-11
HSA-5676594	TNF receptor superfamily (TNFSF) members, NF-kB pathway	1.59e-08
hsa04672	Intestinal immune network for IgA production	1.47e-07
hsa04064	NF-kappa B signaling pathway	1.95e-06
hsa04060	Cytokine-cytokine receptor interaction	7.33e-05
HSA-5669034	TNFs bind their physiological receptors	3.89e-05
hsa05340	Primary immunodeficiency	0.0049
hsa04622	RIG-I-like receptor signaling pathway	0.0133
hsa04657	IL-17 signaling pathway	0.0189
hsa05222	Small cell lung cancer	0.0189

Functional enrichments in Network 16

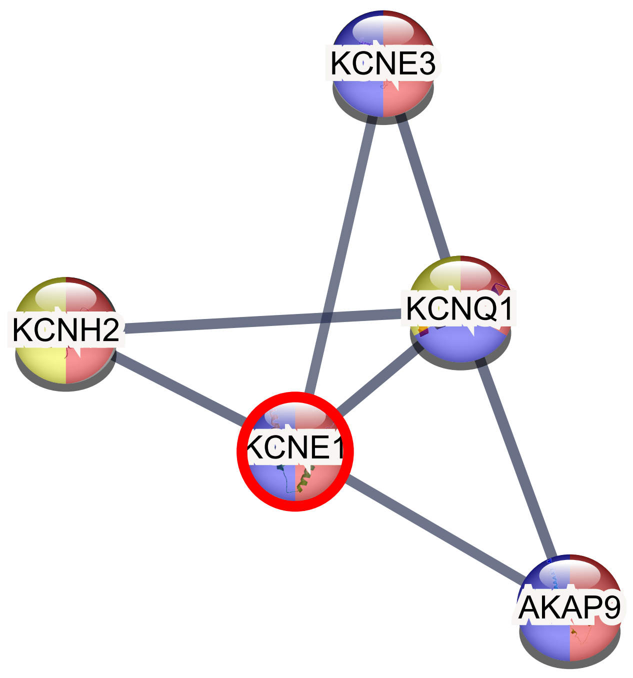

Pathways	Description	False discovery rate
HSA-5576890	Phase 3 - rapid repolarisation	1.17e-13
HSA-5576893	Phase 2 - plateau phase	3.44e-09
HSA-1296072	Voltage gated Potassium channels	0.0224

Functional enrichments in Network 17

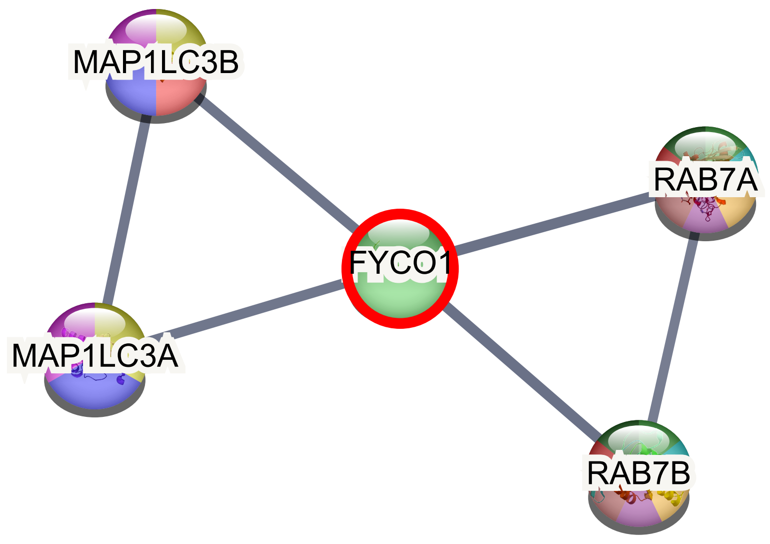

Pathways	Description	False discovery rate
HSA-8854214	TBC/RABGAPs	0.00028
HSA-8934903	Receptor mediated mtophagy	0.0029
hsa05132	Salmonella infection	0.0041
HSA-5205685	PINK1-PRKN Mediated Mitophagy	0.0078
hsa04216	Ferroptosis	0.0079
hsa04137	Mitophagy - animal	0.0121
hsa05146	Amoebiasis	0.0224
hsa04140	Autophagy - animal	0.0299
hsa04145	Phagosome	0.0299
HSA-8873719	RAB geranylgeranylation	0.0403

Functional enrichments in Network 18

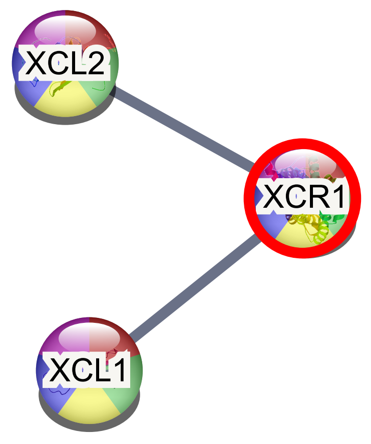

Pathways	Description	False discovery rate
hsa04061	Viral protein interaction with cytokine receptor	4.22e-05
HSA-380108	Chemokine receptors bind chemokines	5.95e-05
hsa04062	Chemokine signaling pathway	0.00015
hsa04060	Cytokine-cytokine receptor interaction	0.00034
HSA-416476	G alpha (q) signalling events	0.0011

Functional enrichments in Network 19

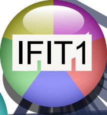
IFIT1

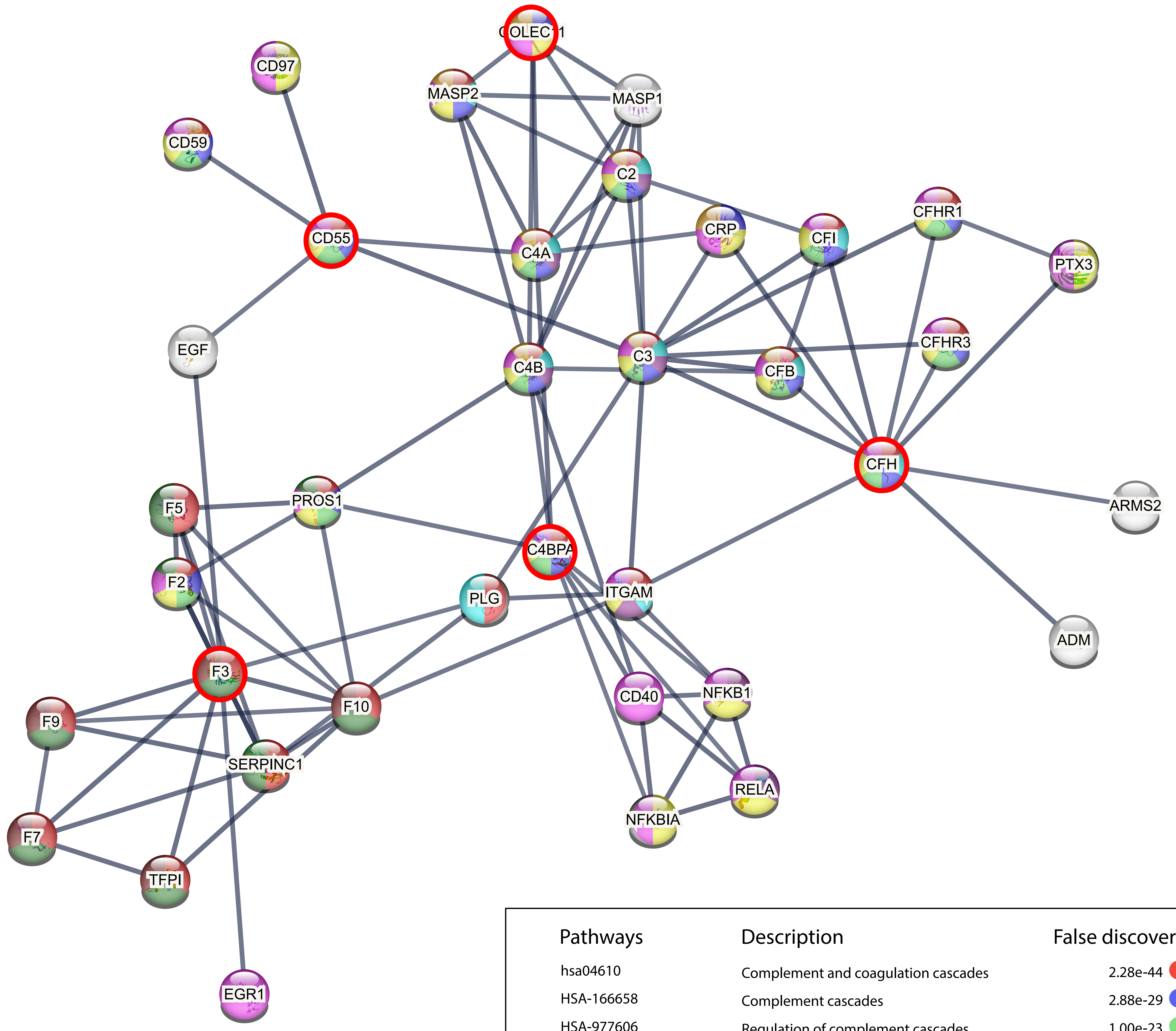

Pathways	Description	False discovery rate
hsa04610	Complement and coagulation cascades	2.28e-44
HSA-166658	Complement cascades	2.88e-29
HSA-977606	Regulation of complement cascades	1.00e-23
HSA-168249	Innate immune system	9.50e-18
HAS-168256	Immune system	3.40e-14
HSA-140877	Formation of fibrin clot (Clotting cascade)	7.33e-14
HSA--166663	Initial triggering of complement	1.47e-13
hsa05150	Staphylococcus aureus infection	2.57e-13
hsa05133	Pertussis	2.59e-10
HSA-140834	Extrinsic pathway of fibrin clot formation	1.40e-09

Functional enrichments in Network 2

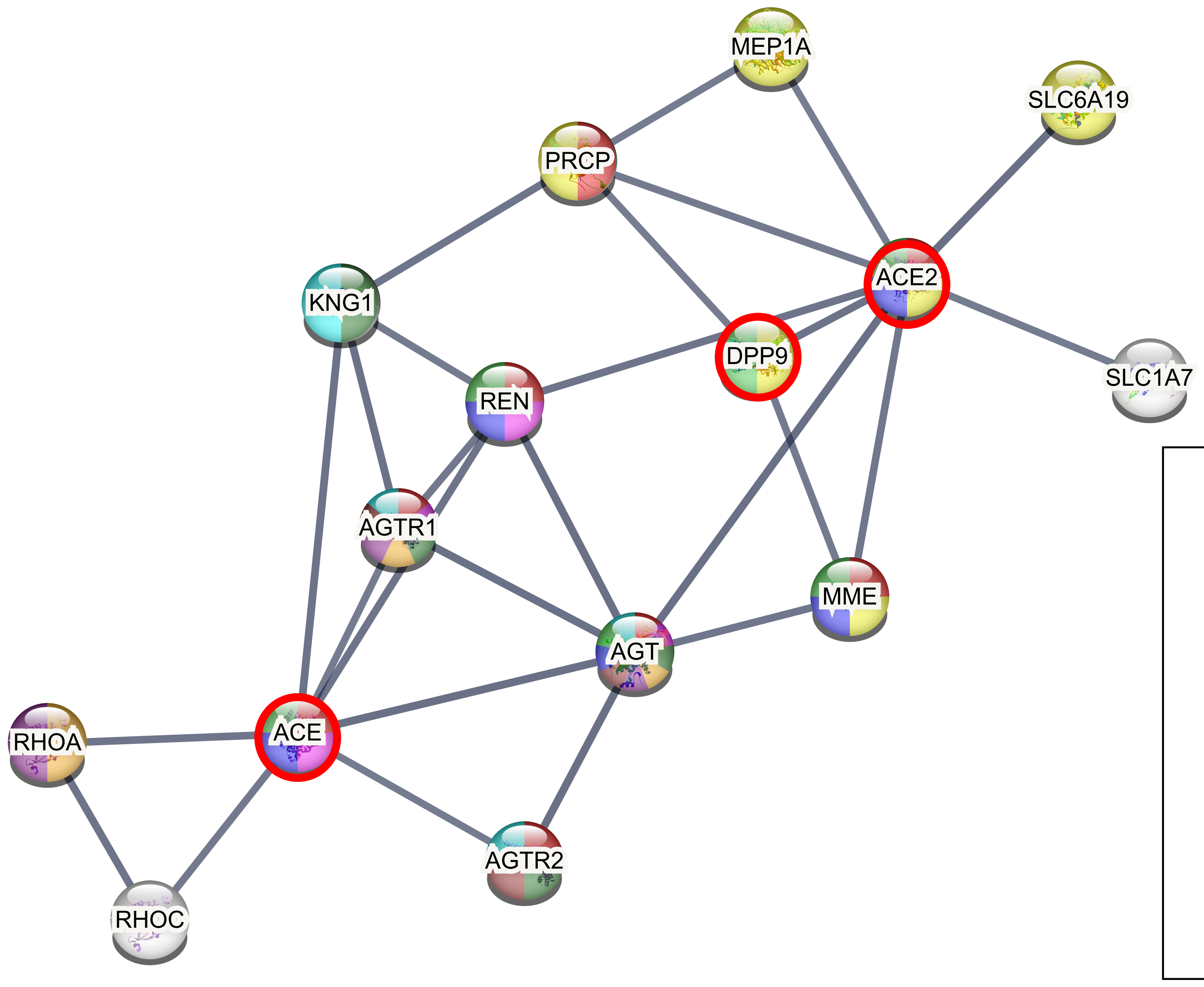

Pathways	Description	False discovery rate
hsa04614	Renin-angiotensin system	3.16e-17
HSA-2022377	Metabolism of Angiotensinogen to angiotensins	9.08e-09
HSA-2980736	Peptide hormone metabolism	5.12e-08
hsa04974	Protein digestion and absorption	1.76e-08
hsa04924	Renin secretion	2.22e-05
hsa04080	Neuroactive ligand-receptor interaction	0.0082
HSA-375276	Peptide ligand-binding receptors	0.0090
hsa04270	Vascular smooth muscle contraction	0.0094
hsa04072	Phospholipase D signaling pathway	0.0105
hsa04261	Adrenergic signaling in cardiomyocytes	0.0105

Functional enrichments in Network 3

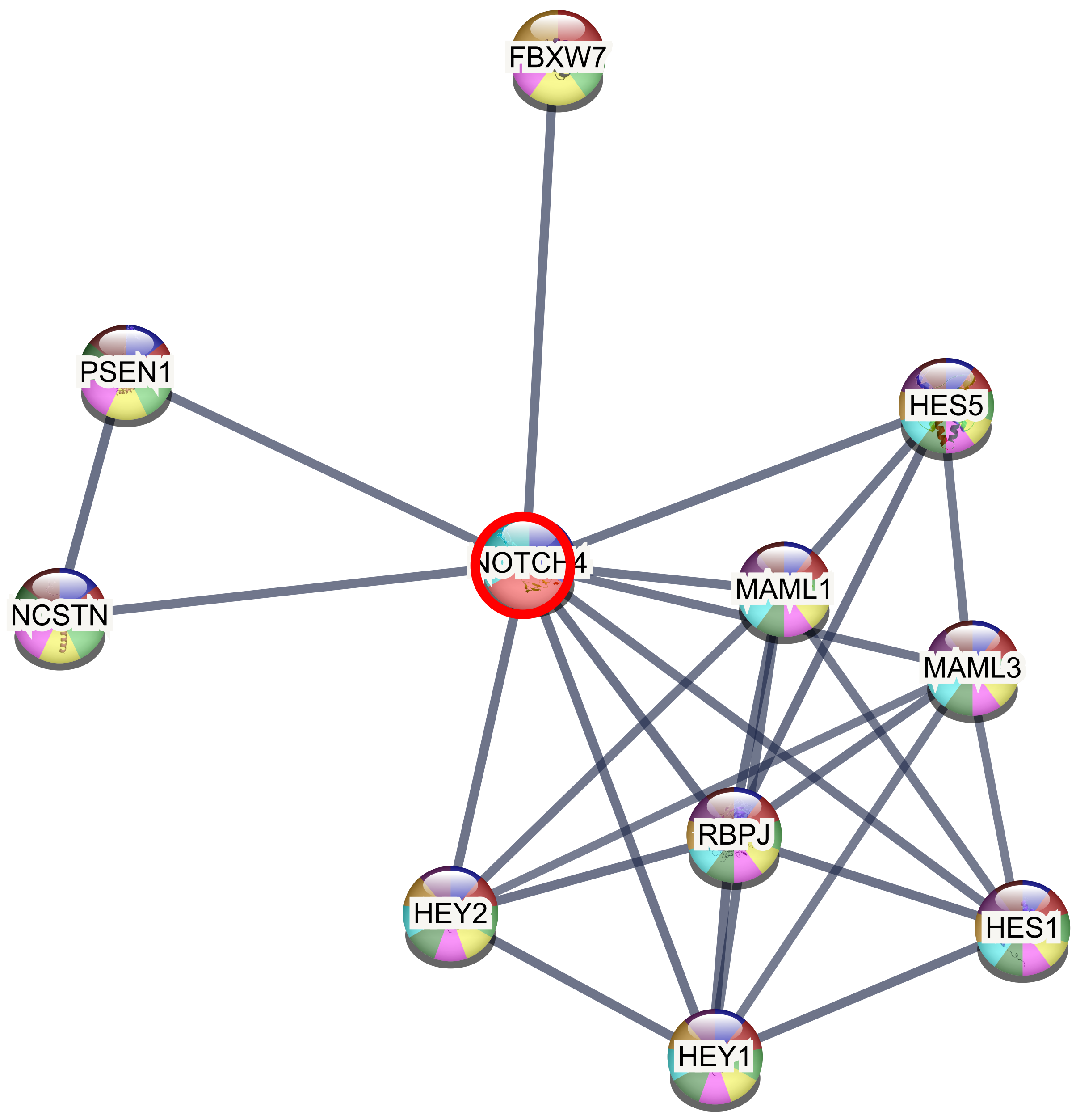

Pathways	Description	False discovery rate
HSA-9013694	Signaling by NOTCH4	2.54e-23
has04330	NOTCH signaling pathway	1.74e-22
HSA-2644606	Constitutive signaling by NOTCH1 PEST domain mutants	1.52e-21
HSA-2894862	Constitutive signaling by NOTCH1 HD+PEST domain mutats	152e-21
HSA-1980143	Signaling by NOTCH1	4.13-e21
HSA-9012852	Signaling by NOTCH3	1.21e-19
HSA-9013695	NOTCH4 intracellular domain regulates transcription	2.42e-26
HSA-2122947	NOTCH1 intracellular domain regulates transcription	8.60e-17
HSA-9013508	NOTCH3 intracellular domain regulates transcription	9.16e-16
HSA-1980145	Signaling by NOTCH2	4.68e-15

Functional enrichments in Network 4

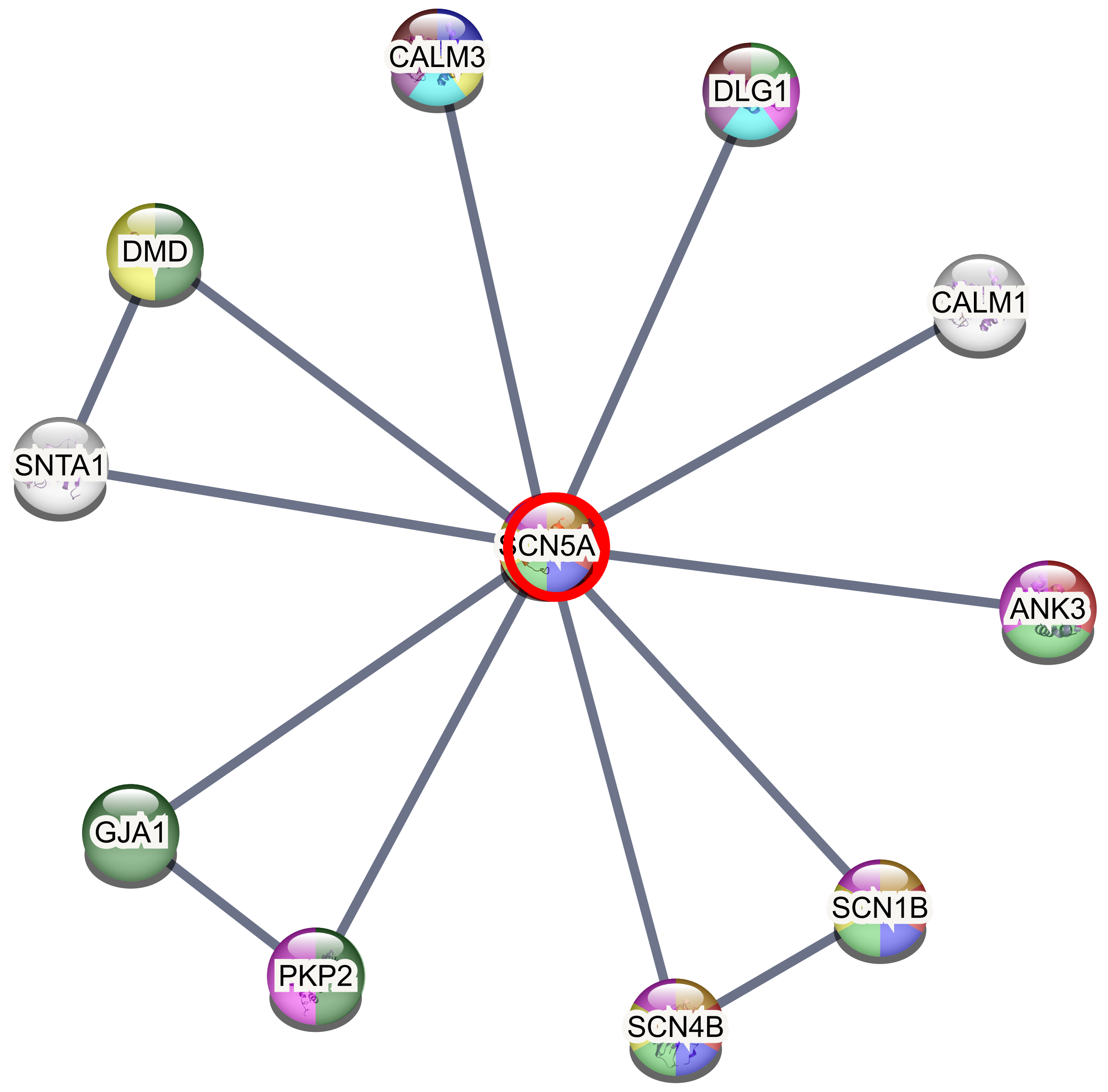

Pathways	Description	False discovery rate
HSA-445095	Interaction btween L1 and Ankyrins	6.08e-06
HSA-5576892	Phase 0 -rapid depolarisation	6.08e-06
HSA-373760	L1CAM interactions	6.08e-06
HSA-397014	Muscle contraction	2.40e-05
HSA-397014	Developmental biology	0.0029
hsa05412	Arrhythmogenic right ventricular cardiomyopathy	0.0034
HSA-451308	Activation of Ca-permeable Kainate receptor	0.0046
hsa04261	Adrenergic signaling in cardiomyocytes	0.0117
HSA-442982	Ras activation upon Ca2+ influx throught NMDA	0.0130
HSA-438066	Unblocking of NMDA receptors, glutamate binding	0.0131

Functional enrichments in Network 5

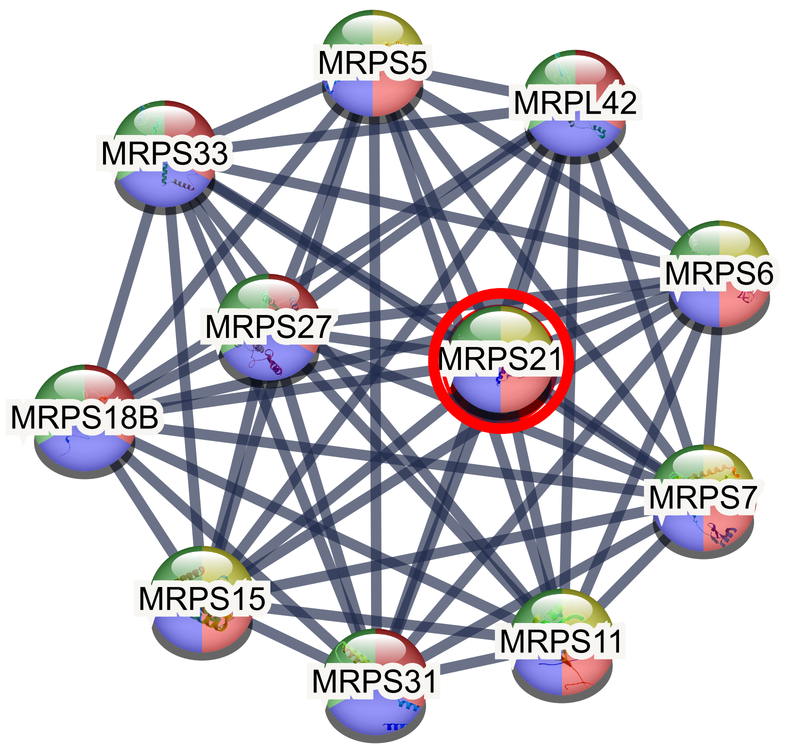

Pathways	Description	False discovery rate
HSA-5368286	Mitochondrial translation initiation	6.77e-23
HSA-5389840	Mitochondrial translation elongation	6.77e-23
HSA-5419276	Mitochondrial translation termination	6.77e-23
hsa03010	Ribosome	1.52e-08

Functional enrichments in Network 6

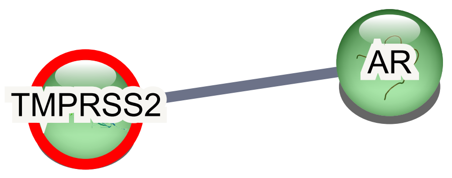

Pathways	Description	False discovery rate
hsa05215	Prostate cancer	0.0083

Functional enrichments in Network 20

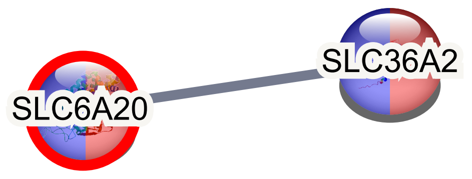

Pathways	Description	False discovery rate
HSA-352230	Amino acid transport across the plasma membrane	0.0068
HSA-5619102	SLC transporter disorders	0.0286

Functional enrichments in Network 21

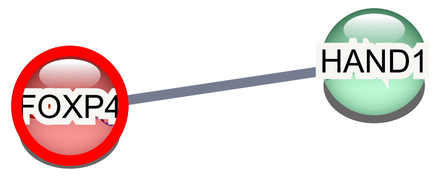

Pathways	Description	False discovery rate
N.A	No significant pathway related to Network 22	

Functional enrichments in Network 22

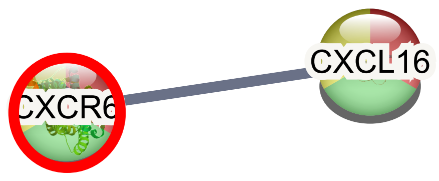

Pathways	Description	False discovery rate
HSA-380108	Chemokine receptors bind chemokines	3.53e-02
hsa04062	Chemokine signaling pathway	3.09e-02
hsa04060	Cytokine-cytokine receptor interaction	3.53e-02

Functional enrichments in Network 23

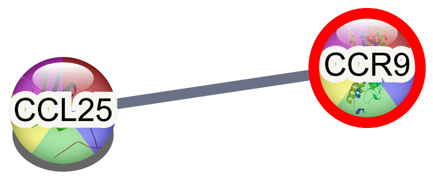

Pathways	Description	False discovery rate
hsa04672	Intestinal immune network for IgA production	1.70e-03
hsa04061	Viral protein interaction with cytokine receptor	4.20e-03
hsa04062	Chemokine signaling pathway	1.03e-02
hsa04060	Cytokine-cytokine receptor interaction	1.76e-02
HSA-380108	Chemokine receptors bind chemokines	1.94e-02

Functional enrichments in Network 24
